# Antigen-directed single domain antibody-based TNFR1 agonists elicit preferential killing of HER2-overexpressing cancer cells

**DOI:** 10.1016/j.isci.2026.115327

**Published:** 2026-03-11

**Authors:** Laura Unmuth, Britta Lipinski, Alicia Hoerr, Julia Harwardt, Enrico Guarnera, Michal Szczepek, Stefan Becker, Andreas Menrad, Patrick Scheerer, Andreas Evers, Simon Krah, Desislava Elter, Lukas Pekar, Stefan Zielonka

**Affiliations:** 1 Biomolecular Immunotherapy, Institute for Organic Chemistry and Biochemistry, Technical University of Darmstadt, 64287 Darmstadt, Germany; 2 Antibody Discovery and Protein Engineering, Merck Healthcare KGaA, 64293 Darmstadt, Germany; 3 Charité – Universitätsmedizin Berlin, Corporate Member of Freie Universität Berlin, Humboldt-Universität zu Berlin, Institute of Medical Physics and Biophysics, Group Structural Biology of Cellular Signaling, 10117 Berlin, Germany

**Keywords:** molecular biology, immunology, cancer

## Abstract

In this study, we present a strategy to uncouple tumor necrosis factor (TNF)-like cell death induction from TNFR2 agonism in a tumor-targeted fashion. Single-domain antibodies (sdAbs) targeting TNFR1 were generated by combining camelid immunization with yeast surface display. Reformatting of resulting paratopes as bispecific antibodies (bsAbs) in a 2 + 2 manner by employing an sdAb-based paratope targeting HER2 revealed the identification of an immunocytokine-like bsAb, referred to as immunocytokine mimetic (ICM), which triggered TNF-like tumor cell death of HER2-overexpressing cancer cells as well as robust caspase-1, -3, and -8 activation in a *cis*-targeted manner. By modulating the valency of the TNFR1-directed sdAb, killing capacities as well as caspase activities of HER2-targeted ICMs were significantly augmented, eventually resulting in enhanced cell death induction when compared with TNF. Moreover, HER2-targeted TNFR1 ICMs also displayed a beneficial, i.e., substantially reduced profile in inducing unconditional pro-inflammatory cytokine release from peripheral blood mononuclear cells (PBMCs).

## Highlights

• HER2-targeted TNFR1 agonists induce preferential cell death of HER2+ tumor cells
• HER2-targeted TNFR1 immunocytokine mimetics activate caspase-1, -3, and -8
• TNFR1 paratope valency engineering enhances tumor cell death and caspase activation
• HER2-targeted TNFR1 agonists decouple cell killing from NF-κB signaling

## Graphical abstract

**Figure:**
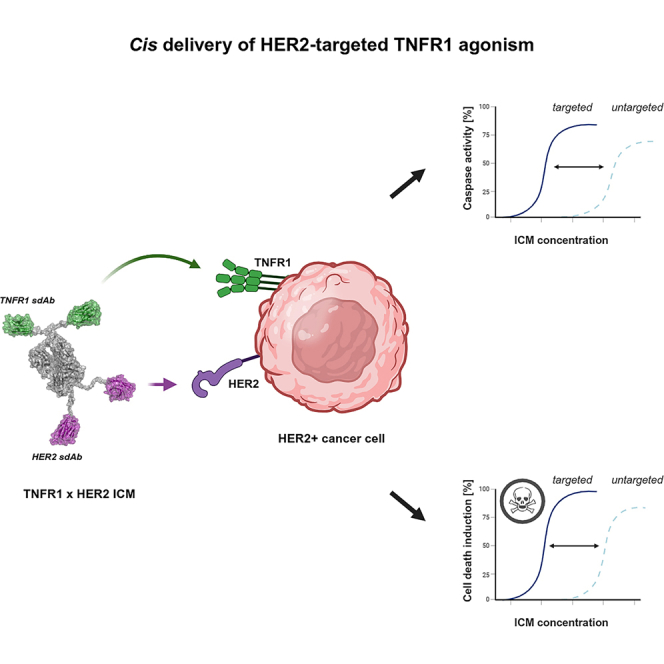

## Introduction

Tumor necrosis factor (TNF) is one of the main mediators of inflammatory processes. As a pro-inflammatory cytokine, TNF is capable of eliciting cell death, immune cell activation, and inflammation.^1^ TNF is primarily produced by activated macrophages and lymphocytes, but also by non-immune cells such as endothelial cells and fibroblasts.^2^^,^^3^ TNF exists in two forms, a membrane-bound form and a trimeric soluble form, which is generated through proteolytic cleavage by a metalloprotease referred to as TNF-alpha converting enzyme (TACE).^4^^,^^5^ TNF exerts its function by binding to two distinct receptors, TNFR1 and TNFR2. TNFR1 is ubiquitously expressed, while TNFR2 is primarily found on the immune cell compartment. The capability of triggering cell death is primarily mediated by the interaction of TNF with TNFR1. Binding of TNF to TNFR1 might elicit apoptosis in a caspase-8-dependent manner, but also pyroptosis as well as necroptosis have been described as mechanisms of TNF-derived cell death.^6^ In addition, TNFR1 agonism might trigger the release of pro-inflammatory mediators such as cytokines and chemokines, as well as pro-survival signaling.^6^ The functional ramifications of TNF binding to TNFR2 are cell type and context-dependent.^1^ In this regard, TNFR2 agonism on NK cells can promote their activation, cytotoxicity, and interferon-γ (IFN-γ) secretion.^7^ However, chronic TNFR2 stimulation of NK cells might result in the upregulation of immune checkpoints. On conventional T cells, TNFR2 functions as a co-stimulatory receptor, but it might also facilitate activation-induced cell death.^8^ In addition to this, TNFR2 is highly expressed on regulatory T cells (T_regs_), where it is critical for T_reg_ proliferation and survival.^9^^,^^10^^,^^11^ Furthermore, TNFR2 agonism on myeloid-derived suppressor cells (MDSCs) stimulates their activation and infiltration into the tumor microenvironment.^12^ As such, TNF is a cytokine that is inherently pleiotropic in its nature.

TNF has been exploited as a potential therapeutic agent for the treatment of cancer.^13^ Beromun, a recombinantly expressed version of human TNF, has been approved in Europe for metastatic melanoma and soft tissue sarcoma.^14^^,^^15^ However, due to the narrow therapeutic window of recombinant human (rh) TNF,^16^ next-generation cytokine-like molecules, named immunocytokines, have been engineered that exploit TNF as a payload fused to a tumor-associated antigen-targeted paratope for the preferential accumulation at the site of the disease.^17^^,^^18^ In this regard, Neri and colleagues generated an immunocytokine that combines TNF with an antibody derivative that delivers the cytokine to the extracellular matrix of tumors by targeting the alternatively spliced extradomain B of fibronectin.^19^ This compound, referred to as Fibromun, has demonstrated promising efficacy in different preclinical settings.^20^^,^^21^^,^^22^^,^^23^^,^^24^ Even more importantly, encouraging results were obtained from Phase I clinical trials, especially for patients with soft tissue sarcoma or glioblastoma, and additional clinical studies are underway.^25^^,^^26^^,^^27^ In addition, Fibromun is currently being investigated in clinical trials in combination with the same antibody paratope fused to an IL-2 payload for the treatment of various types of skin cancers. This immunocytokine combination, called Nidlegy, met the primary endpoint in a phase III study as neoadjuvant treatment for patients with locally advanced melanoma.^28^

Besides exploiting TNF for preferential tumor delivery, TNFR2 receptor modulation emerged as a promising strategy for cancer treatment.^29^ Antibody-based TNFR2 antagonism is being exploited to block the immunosuppressive functions of Tregs and MDSCs, which can be combined with the depletion of these cells via antibody-dependent cell-mediated cytotoxicity (ADCC) or antibody-dependent cellular phagocytosis.^30^^,^^31^ On the other hand, TNFR2 agonism to provide co-stimulation of T cells and NK cells is currently being scrutinized.^32^^,^^33^^,^^34^ In contrast to this, receptor modulation of TNFR1 has not been explored excessively for cancer therapy. In one of the few studies published, Kim et al. generated a TNF-derived mutant preferentially binding to TNFR1, which was fused to Obinutuzumab.^35^ The resulting immunocytokine elicited stronger cell killing of CD20-overexpressing B cell lymphoma cells than Obinutuzumab alone.

**Figure 1:**
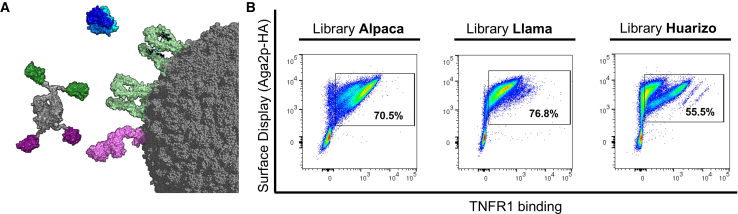
Generation of targeted immunocytokine mimetics (ICMs) in a bispecific bivalent camelid-derived sdAb-based architecture after yeast surface screening (A) Trimeric (rh) TNF (blue) triggers trimeric TNFR1 (light green) downstream signaling. Bispecific bivalent ICMs are able to elicit trimeric TNFR1 downstream signaling by binding of HER2-targeting sdAbs (purple) to HER2 (light purple) and engaging trimeric TNFR1 (light green) with camelid-derived TNFR1-targeting paratopes (green). Structural visualization was generated with PyMOL software version 2.3.0, based on PDB entries 7K7A and 1TNF and structural modeling as described in the STAR Methods section. (B) Enrichment after three sorting rounds of each library against (rh) TNFR1 ECD. A two-dimensional sorting strategy was applied to detect full-length VHH display simultaneous to antigen binding at a concentration of 1 μM. Plots show 5 × 10^4^ events of the corresponding sorting output and the percentage of gated cells to visualize enrichment.

Within the present study, we set out to investigate whether TNFR1 agonism can be harnessed for preferential tumor cell killing. To this end, camelid-derived single-domain antibodies (sdAbs) were isolated from immunized animals by yeast surface display.^36^^,^^37^ TNFR1-specific sdAbs were *N*-terminally grafted onto the hinge region of an effector-silenced IgG1 Fc region that harbored a HER2-specific sdAb paratope at the *C-*terminus, resulting in bivalently targeting both, TNFR1 and HER2 (2 + 2, Figure 1A). Among those bispecifics, agonists were identified in TNF-reporter cell assays and further assessed on HER2-expressing MCF-7 cancer cells, revealing differential induction of cell death by a subset of engineered immunocytokine mimetics (ICMs). One particular ICM elicited robust TNF-like cancer cell lysis, which was clearly HER2-dependent. Moreover, we demonstrate that by modulating the valency of the TNFR1-targeting paratope, killing capacities can be significantly augmented, resulting in ICMs that were more potent in facilitating cancer cell death as well as in caspase-1, -3, and -8 activation than the wild-type cytokine TNF. Ultimately, this study provides evidence that sdAb-derived TNFR1 ICMs might be valuable tools for *cis*-targeting of tumor cells, eventually resulting in cell death induction, which can be decoupled from NF-κB pro-survival signaling.

## Results

### Generation of camelid-derived sdAbs targeting TNFR1

A llama (*Lama glama*), an alpaca (*Vicugna pacos*), and a huarizo (*Lama glama* x *Vicugna pacos*) were immunized four times with rh TNFR1 extracellular domain (ECD). After yeast surface display VHH library generation (per specimen), we simultaneously co-selected for (rh) TNFR1 ECD binding as well as full-length VHH display in a two-dimensional manner. Within three rounds of fluorescence-activated sorting (FACS), we were able to enrich for TNFR1-binding populations from all three sub-libraries (Figures 1B and S1). Single clone sequencing after library sorting unveiled 55 unique clones in total. Based on the amino acid diversity of CDR3, 17 clones were chosen for reformatting. To this end, TNFR1-addressing sdAbs were grafted onto the *N*-terminal hinge region of an effector-silenced Fc region. In addition to this, a HER2-targeting VHH^38^ was fused to the *C*-terminus of the Fc-part and separated by a 15 amino acid (3xGly_4_Ser) linker, resulting in bivalent targeting of TNFR1 as well as HER2 (2 + 2, Figure 1A). Antibody production was conducted in Expi293 cells, followed by protein A purification. Besides four variants which we have not been able to produce at all, all remaining 13 bispecific antibodies (bsAbs) showed high purities, i.e., target peaks, as determined by analytical size exclusion chromatography post protein A purification (Table S1). Subsequently, binding experiments were conducted against (rh) TNFR1, (rh) TNFR2 as a negative control antigen, as well as (rh) HER2 for tumor targeting using biolayer interferometry (BLI). To this end, bispecific ICMs were loaded onto the biosensor tips. Antigens were utilized at a concentration of 250 nM. This revealed specific binding of twelve clones against (rh) TNFR1, whereas no binding at all was observed against (rh) TNFR2 (Figure S2A). Furthermore, all molecules showed similar binding to (rh) HER2, ultimately demonstrating adequate specificities of generated ICMs (Figure S2B).

### A subset of TNFR1xHER2-targeting ICMs is capable of agonizing TNFR1 in reporter cells

**Figure 2:**
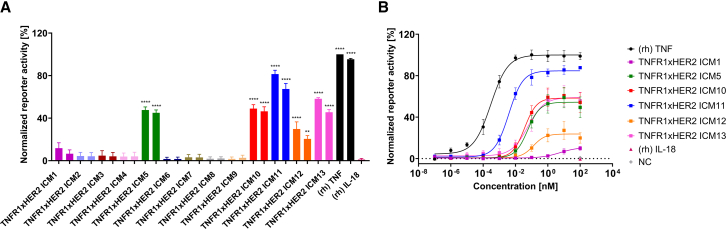
TNFR1-and HER2-specific bispecific bivalent ICMs (2 + 2) trigger reporter cell activity (A) HEK-Blue TNF reporter cells were incubated with two fixed concentrations of 10 nM and 1 nM of bsAbs, as well as 0.1 nM and 0.01 nM of (rh) TNF as a positive control and 0.1 nM of (rh) IL-18 as a negative control. After 24 h of incubation, secreted embryonic alkaline phosphatase activity was measured via OD_640_. Reporter activity was normalized to (rh) TNF signal. (B) Dose-titration of agonistic bsAbs in HEK-Blue TNF reporter cells. Mean values ±SEM of three independent experiments are shown. ∗∗∗∗*p* < 0.0001,∗∗∗*p* < 0.001, ∗∗*p* < 0.01, and ∗*p* < 0.05 calculated by utilizing one-way ANOVA analyses and Bonferroni test.

All twelve TNFR1xHER2-targeting bispecific ICMs were assessed with respect to their agonistic potential by harnessing TNF reporter cells, stably expressing TNFR1 as well as TNFR2 (HEK-Blue TNF cells). Initially, bsAbs were exploited at two fixed concentrations of 10 nM and 1 nM. (rh) TNF was used as a positive control at concentrations of 0.1 nM as well as 0.01 nM, and (rh) IL-18 was harnessed as a negative control at 0.1 nM. As expected, (rh) TNF enabled a robust activation of the reporter cells at both concentrations, whereas no significant agonism was observed for (rh) IL-18 (Figure 2A). Out of the twelve bsAbs investigated, six ICMs induced reporter activation of more than 10% compared with TNF at a higher concentration of 10 nM. In this regard, identified TNFR1 agonists were strikingly different in their ability to trigger reporter cell activation, ranging from weak agonism at a concentration of 10 nM for ICM1 of 10.9% (normalized to (rh) TNF) to quite strong TNFR1 activation for ICM11 of 84.7% at 10 nM.

All six ICMs eliciting significant agonism were further investigated in terms of their agonism in a dose-dependent manner by exploiting the same reporter cell assay (Figure 2B). In this regard, (rh) TNF was potent in triggering reporter cell activation, with an EC_50_ of 0.3 pM. Out of the six ICMs, five bispecifics showed dose-dependent activation of the reporter cells. ICM1 only triggered marginal TNFR1 activation at high concentrations and was consequently excluded from further characterization. Of note, we observed substantial differences in potencies (EC_50_ of reporter cell activation) as well as efficacies (normalized E_max_ of agonism compared to (rh) TNF) for the five remaining ICMs (Table S1). While ICM11 strongly elicited reporter cell activation (EC_50_ = 4 pM, E_max_ = 84.7%), ICM12 was heavily attenuated with potencies of 127 pM and a maximal activation of 23.7% compared to (rh) TNF. ICMs 4, 10, as well as 13, were moderate agonists with potencies in the double-digit pM range and efficacies of 54.4–59.0% when compared with (rh) TNF.

Importantly, differences in affinities for targeting TNFR1 were not primarily responsible for the divergence in agonism, since BLI experiments revealed binding in the lower double-digit nanomolar range for all five ICMs (Figure S3A; Table S2). Also, epitope binning experiments unveiled similar, presumably immunodominant epitopes on TNFR1 that were addressed by the investigated bsAbs (Figure S3B). In this respect, all ICMs also shared the same bin with (rh) TNF, indicating that the natural binding site of the wild-type cytokine seems to be privileged for agonism.

Since HEK-Blue cells naturally express HER2 on the surface (Figure S4A), we also set out to explore a *cis*-targeting effect of the generated TNFR1xHER2 ICMs. In detail, we wanted to see whether blocking HER2 on the surface of HEK-Blue cells would result in attenuated potencies of generated molecules. To this end, reporter cells were pre-incubated with an excess of Trastuzumab (1 μM) before ICM addition, because Trastuzumab, as well as the exploited VHH-derived paratope, target the same or an overlapping epitope on HER2 (Figure S2C). Interestingly, HER2 blocking by Trastuzumab resulted in significantly attenuated potencies for all ICMs tested (Figure S5; Table S1), clearly indicating HER2-dependency as well as *cis*-delivery of TNFR1 agonism.

### TNFR1xHER2-targeting ICM elicits TNF-like cell death induction of HER2-expressing cancer cells

**Figure 3:**
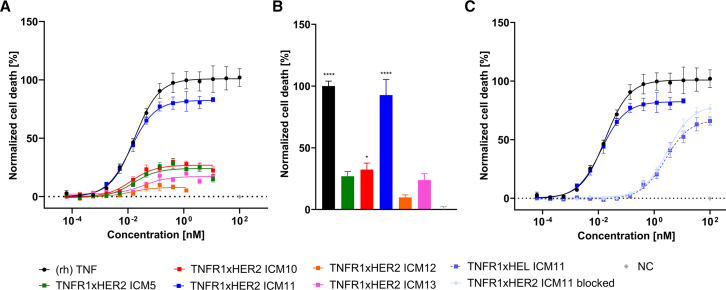
TNFR1xHER2 targeting ICMs induce TNF-like cell death of HER2 expressing MCF-7 breast cancer cells in a *cis*-targeted manner (A) MCF-7 cells were stimulated with increasing concentrations of five leading TNFR1xHER2 ICMs and (rh) TNF for 96 h. Killing was monitored by green fluorescence signal with SYTOX Green Dead Cell Stain. Killing signal was normalized to (rh) TNF signal. (B) Cell death induction of different TNFR1xHER2 ICMs normalized to (rh) TNF. All compounds were applied at a concentration of 1 nM for 96 h (C) Evaluation of *cis*-targeting in the cell death induction of MCF-7 cells. Instead of the HER2-directed paratope, a hen egg lysozyme (HEL)-specific VHH was incorporated at the *C*-terminus of ICM11. Additionally, cell death induction of TNFR1xHER2 ICM was measured after the pre-incubation of MCF-7 cells with 1 μM Trastuzumab. Mean values ±SEM of three independent experiments are shown. ∗∗∗∗*p* < 0.0001,∗∗∗*p* < 0.001, ∗∗*p* < 0.01, and ∗*p* < 0.05 calculated by utilizing one-way ANOVA analyses and Bonferroni test.

Next, we aimed at investigating whether TNFR1xHER2-targeting ICMs can induce preferential cell death of HER2-overexpressing cancer cells. For this, we identified HER2-expressing human breast cancer cell line MCF-7 as reasonably sensitive to (rh) TNF-treatment (Figure S4B; Figure S6). We compared killing capacities of the five TNFR1xHER2-targeting ICMs to (rh) TNF (Figure 3A; Table 1). ICM11, which robustly induced reporter cell activation, also solidly elicited cell death of MCF-7 cells (EC_50_ = 12.5 pM, E_max_ normalized to TNF = 82.4%). In contrast to this, the other four ICMs only marginally triggered killing of this cell line, with maximal killing capacities ranging between 8.0% for ICM12 and 26.9% for ICM10 in comparison to (rh) TNF treatment (Figure 3B). Of note, at higher - potentially physiological irrelevant - concentrations we witnessed a hooking effect for generated ICMs (Figure S7). This phenomenon has been described previously for bispecific agonistic antibodies.^39^^,^^40^^,^^41^^,^^42^

**Table 1:** Biophysical, biochemical, and functional killing properties of HER2xTNFR1 surrogate agonists

Samples	Purity [%]	Yield [mg/L]	MCF-7 killing	
			EC_50_ [pM]	E_max_ [% to TNF]
TNFR1xHER2 ICM5	95.1	83.9	15.6	24.1
TNFR1xHER2 ICM10	95.8	83.6	13.1	26.9
TNFR1xHER2 ICM11	96.8	102.3	12.5	82.4
TNFR1xHER2 ICM12	98.8	127.7	12.4	8.0
TNFR1xHER2 ICM13	96.5	96.2	37.7	17.2
TNFR1xHEL ICM11	100	125.4	2514	67.1
TNFR1xHER2 ICM11 blocked	–	–	2923	78.8
TNFR1xHER2 ICM11 (4 + 2)	98.2	36.4	1.3	95.5
TNFR1xHER2 ICM11 (6 + 2)	96.7	24.2	1.5	111.9
TNFR1xHEL ICM11 (4 + 2)	98.8	67.8	30.9	88.8
TNFR1xHEL ICM11 (6 + 2)	98.5	49.9	8.8	101.3
(rh) TNF	–	–	17.6	100

Subsequently, we focused on ICM11 for further characterization, since this molecule induced TNF-like killing of MCF-7 cells. ICM11 binds to (rh) TNFR1 with affinities of 15.7 nM (Table S2). We also tested the complex formation of ICM11 and (rh) TNFR1 in an analytical SEC setup (Figure S8). TNFR1 and ICM11 each eluted as single, separate peaks, whereas the pre-incubated mixture produced a distinct, earlier peak, consistent with complex formation. SDS-PAGE of fractions B1–B12 confirmed the co-elution of TNFR1 and ICM11 in the complex fractions. These results were reproducible across three independent runs and provide clear biochemical evidence of a stable (rh) TNFR1-ICM11 complex and hence, robust binding of ICM11 to (rh) TNFR1**.**

### Killing capacities of TNFR1xHER2-specific ICMs can be augmented by antibody engineering

Having established both biochemical complex formation and a TNF-like epitope for ICM11 on TNFR1 (Figure 7; Figure S8), we next scrutinized whether the induction of cell death depends on HER2 expression (Figure 3C). To this end, we produced a hen egg lysozyme (HEL)-targeting derivative of ICM11 as a negative control (TNFR1xHEL ICM11). Whereas HER2-targeting ICM11 mediated potent killing of MCF-7 cells similar to (rh) TNF, the potencies of the TNFR1xHEL ICM11 control molecule were substantially attenuated (EC_50_ = 2.51 nM). In addition, blockade of HER2-binding by pre-incubation with an excess (1 μM) of Trastuzumab attenuated the potencies of TNFR1xHER2 ICM11 in a very similar manner (EC_50_ = 2.92 nM). Essentially, delivering TNFR1 agonists in *cis* using TNFR1xHER2 ICM11 resulted in more than 200-fold higher potencies, clearly highlighting the impact of HER2 as a tumor anchor.

**Figure 4:**
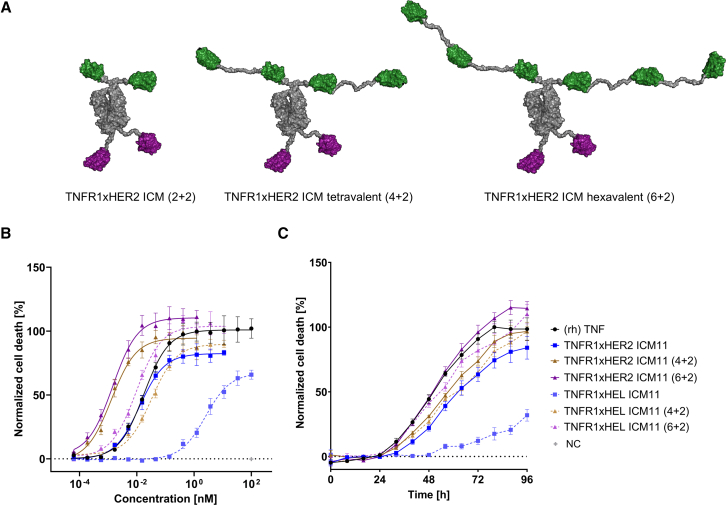
Engineering of TNFR1 paratope valencies enables augmented cell death induction of MCF-7 cells (A) Schematic depiction of valency-engineered TNFR1xHER2 targeting ICMs. A TNFR1-targeting VHH is fused *N*-terminally to the hinge region of an immune effector-silenced IgG1 Fc. A HER2-targeting VHH is fused *C-*terminally by employing a 15 amino acid linker (3xGly_4_Ser), resulting in the initially generated (2 + 2) format. To increase valencies of TNFR1, multivalent constructs were designed by linking TNFR1-targeting VHHs in tandem for tetravalent (4 + 2) or in tridem for hexavalent (6 + 2) targeting of TNFR1 and bivalent binding to HER2. All building blocks were separated by a 15 amino acid linker (3xGly_4_Ser). (B) TNFR1xHER2 multivalent formats were scrutinized for their killing capacities on HER2 expressing MCF-7 cells in comparison to their corresponding TNFR1xHEL negative control ICMs and (rh) TNF. Killing was monitored by green fluorescence signal with SYTOX Green Dead Cell Stain after 96 h of incubation. Killing signal was normalized to (rh) TNF signal. (C) Kinetics of MCF-7 cell death induction by (rh) TNF, bivalent TNFR1xHER2 ICM11 (2 + 2) as well as tetravalent TNFR1xHER2 ICM11 (4 + 2) and hexavalent TNFR1xHER2 ICM11 (6 + 2). Mean values ±SEM of four independent experiments are shown.

In addition, we wanted to enhance the killing capacities of the generated TNFR1xHER2 ICM11 by increasing the valency for TNFR1-targeting. Naturally, soluble TNF forms a trimer, resulting in efficient assembly of the receptor-ligand network and potent downstream signaling. In contrast to this, TNFR1xHER2-directed ICMs were designed to allow for bivalently targeting TNFR1 only (as well as HER2). In order to address the impact of multivalent TNFR1 targeting, we fused the TNFR1-specific paratope of ICM11 in tandem to the hinge region of the effector-silenced Fc (also incorporating the HER2-targeted VHH at the *C-*terminus). Both identical TNFR1-specific sdAbs were separated by a 15 amino acid (3xGly_4_Ser) linker. This resulted in tetravalent targeting of TNFR1 in a 4 + 2 manner (Figure 4A). In addition, we also produced a variant harboring a tridem arrangement of the TNFR1-directed paratope, enabling hexavalent TNFR1 binding in a 6 + 2 manner (Figure 4A). For both molecules, we also expressed the HEL-targeting control entities, allowing for the evaluation of preferential killing of HER2-expressing MCF-7 cancer cells, i.e., an assessment of the *cis*-targeting effect in a quantitative manner (Figures 4B and S7; Table 1). As described above, bivalent targeting of TNFR1 and HER2 by (2 + 2) TNFR1xHER2 ICM11 resulted in TNF-like killing of HER2 overexpressing MCF-7 cells (EC_50_ = 12.5 pM), whereas the unrelated HEL control (2 + 2) was highly attenuated ((2 + 2) TNFR1xHEL ICM11: EC_50_ = 2.51 nM). Consequently, the ratio of HER2-targeting vs. non-targeting (EC_50_) as a quantitative factor for *cis*-engagement was approximately 201. Strikingly, increasing the valency enabling tetravalent TNFR1 binding significantly augmented potencies of eliciting MCF-7 cell death (4 + 2) TNFR1xHER2 ICM11: EC_50_ = 1.3 pM, E_max_ normalized to TNF = 95.5%). Hence, the resulting tetravalent TNFR1xHER2 targeting ICM was more potent in triggering MCF-7 killing than (rh) TNF (EC_50_ = 18 pM). However, also the corresponding HEL-targeting control molecule (4 + 2) TNFR1xHEL ICM11 quite potently triggered lysis of MCF-7 cells (EC_50_ = 30.9 pM), yielding a *cis*-engagement window of the factor of about 23.8. By further increasing the valency of TNFR1 binding to hexavalent targeting (6 + 2), potencies of MCF-7 cell death were not significantly increased ((6 + 2) TNFR1xHER2 ICM11 EC_50_ = 1.5 pM). Yet, we witnessed a trend toward higher maximal killing of 111.9%. Of note, also the corresponding non-tumor-targeted HEL control (6 + 2) TNFR1xHEL ICM11 was augmented in terms of killing potencies (EC_50_ = 8.8 pM), resulting in a diminished factor for *cis*-targeting of approximately 5.9. Essentially, this is giving evidence that the killing capacities of tumor-targeted TNFR1-specific ICMs can be increased by valency engineering. However, this comes with the price of higher untargeted killing, eventually resulting in abated *cis*-delivery of the tailored TNF-like functionality.

We also looked at the kinetics of inducing MCF-7 cell death (Figure 4C). To this end, MCF-7 cells were co-incubated with either (rh) TNF or the valency-engineered ICM11 derivatives at a concentration of 1 nM. Time-resolved killing was monitored every 8 h. For (rh) TNF, we observed the induction of killing starting after 24 h, with roughly half-maximal killing at 48 h and maximum killing achieved after approximately 80 h. In comparison, (2 + 2) TNFR1xHER2 ICM11, as well as (4 + 2) TNFR1xHER2 ICM11, were slightly delayed in eliciting lysis of MCF-7 cells, whereas (6 + 2) TNFR1xHER2 ICM11 triggered cell death with very similar kinetics to (rh) TNF.

### HER2-targeted TNFR1-agonists trigger TNF-like caspase induction in MCF-7 cells

**Figure 5:**
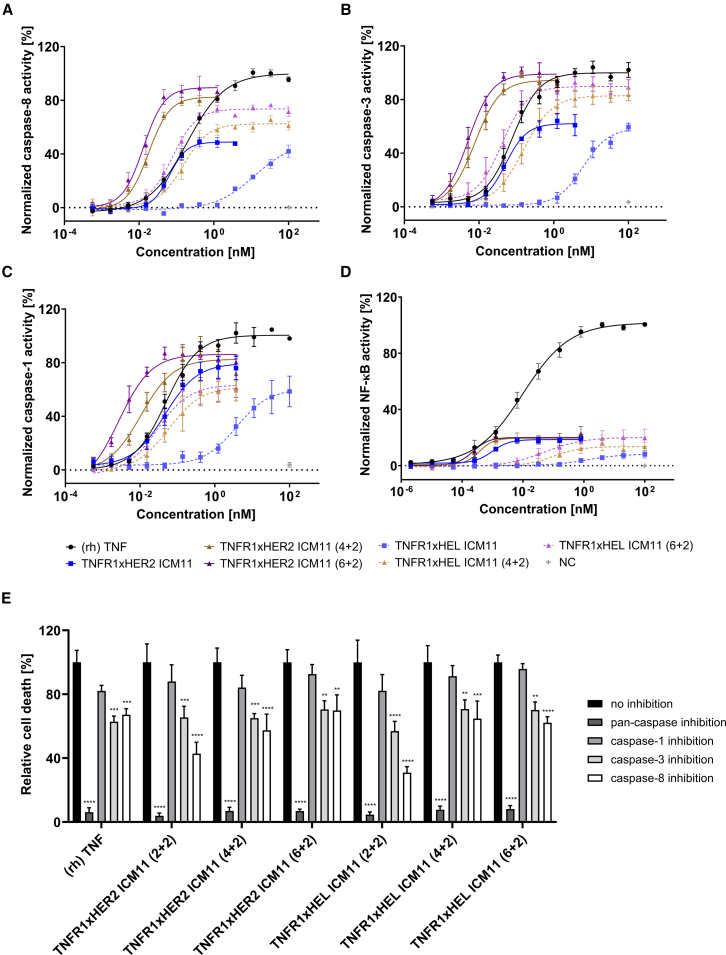
HER2-targeted TNFR1-agonists induce TNF-like caspase-1, caspase-3, and caspase-8 activation decoupled from NF-κB signaling in MCF-7 cells (A–C) Caspase-1/3/8 activation of HER2-expressing MCF-7 cells by ICM11 derivatives compared to (rh)TNF. MCF-7 cells were stimulated with increasing compound concentrations for 72 h. Caspase activities were detected intracellularly with FAM-FLICA(R) Caspase 1 Assay Kit (Biomol), CaspaTag Caspase-3 *In situ* Assay Kit (Merck Millipore), and CaspaTag Caspase-8 *In Situ* Assay Kit (Merck Millipore). Caspase-1/3/8 activation was normalized to (rh) TNF. (D) NF-κB activation in MCF-7 cells triggered by ICM11 derivatives and (rh) TNF. MCF-7 cells were stimulated with increasing concentrations of ICMs and (rh) TNF for 40 min. NF-κB was stained intracellularly with AF488-labeled anti-NF-κB staining antibody (BD) after lysis, fixation, and permeabilization of cells. (E) Remaining relative cell death of MCF-7 cells after treatment with ICM11 derivatives or (rh)TNF in the presence or absence of caspase inhibitors. MCF-7 cells were incubated with caspase-1 inhibitor (InvivoGen), caspase-3 inhibitor (R&D Systems), caspase-8 inhibitor (InvivoGen), or pan-caspase inhibitor (InvivoGen) at 50 μM and a fixed (rh) TNF or ICM concentration of 5 nM for 72 h. Killing was monitored by green fluorescence signal with SYTOX Green Dead Cell Stain and normalized to (rh) TNF signal. Mean values ±SEM of four independent experiments for each figure are shown. ∗∗∗∗*p* < 0.0001,∗∗∗*p* < 0.001, ∗∗*p* < 0.01, and ∗*p* < 0.05 calculated by utilizing two-way ANOVA multiple analyses and Bonferroni test.

It is generally appreciated that TNF exerts its cytotoxic functions by inducing apoptosis.^43^^,^^44^ This form of non-lytic cell death is initiated via caspase-8, which subsequently cleaves and activates executioner caspases, such as caspase-3, to drive cell death.^45^^,^^46^^,^^47^ While both caspase-8 and caspase-3 have traditionally been classified as “apoptotic” caspases, it has been discovered in recent years that both enzymes are also involved in the induction of inflammatory and lytic cell death referred to as pyroptosis.^45^^,^^48^^,^^49^^,^^50^^,^^51^ Very recently, it has been discovered that TNF (in addition to caspase-8 and -3 activation) is capable of driving pyroptosis of breast cancer cells via caspase-1 induction.^45^ To assess whether killing of breast cancer cell line MCF-7 induced by the engineered TNFR1xHER2-specific ICMs is triggered by apoptosis and pyroptosis, we specifically looked at caspase-8, caspase-3, and caspase-1 activation in these cells after ICM treatment. In this regard, (rh) TNF induced caspase-8 activation with potencies in the sub-nanomolar range (Figure 5A; Figure S8; Table 2, EC_50_ = 191 pM). TNFR1xHER2 ICM11 enabling bivalent TNFR1 engagement (2 + 2) triggered caspase-8 activity with similar potencies, albeit to a lower degree (EC_50_ = 64.2 pM, E_max_ normalized to TNF = 48.9%). In line with preferentially induced HER2-targeted killing of MCF-7 cells, the untargeted derivative TNFR1xHER2 ICM11 was highly attenuated, clearly indicating *cis*-targeting (EC_50_ = 12.2 nM, E_max_ normalized to TNF = 49%, attenuated potencies: 191.1-fold). Similar to cell death induction (Figure 4B), the tetravalent (4 + 2) and hexavalent (6 + 2) engineered versions of TNFR1xHER2 ICM11 were significantly more potent in eliciting caspase-8 activity (EC_50_ = 17.8 pM and 13.1 pM, respectively).

**Table 2:** Caspase-1/3/8 and NF-κB activity of bispecific bivalent (2 + 2) and multivalent (4 + 2) and (6 + 2) ICMs in MCF-7 cells

Samples	caspase-1 activation		caspase-3 activation		caspase-8 activation		NF-κB activation	
	EC_50_ [pM]	E_max_ [% to TNF]	EC_50_ [pM]	E_max_ [% to TNF]	EC_50_ [pM]	E_max_ [% to TNF]	EC_50_ [pM]	E_max_ [% to TNF]
TNFR1xHER2 ICM11 (2 + 2)	46.3	79.6	51.2	62.1	64.2	48.9	1.0	18.6
TNFR1xHER2 ICM11 (4 + 2)	10.6	82.7	7.7	94.4	17.8	82.5	0.4	19.6
TNFR1xHER2 ICM11 (6 + 2)	2.7	86.3	6.1	99.0	13.1	89.5	0.2	20.1
TNFR1xHEL ICM11 (2 + 2)	3778	60.5	6136	58.6	12230	49.0	1322	8.3
TNFR1xHEL ICM11 (4 + 2)	58.6	61.9	151.6	82.9	153.2	62.5	102	13.6
TNFR1xHEL ICM11 (6 + 2)	28.4	63.5	41.6	89.8	77.2	73.6	51.2	20.2
(rh) TNF	49.1	100	78.4	100	191.0	100	9.9	100

In line with this, HER2-directed ICMs also robustly triggered the induction of downstream protease caspase-3 (Figure 5B; Table 2). For instance, TNFR1xHER2 ICM11 (2 + 2) drove caspase-3 activation with similar potencies to TNF (EC_50_ = 51.2 pM vs. 78.4 pM, E_max_ normalized to TNF = 62.1%). Again, strong *cis*-targeting was witnessed, since potencies were substantially reduced for the untargeted equivalent TNFR1xHEL ICM11 (2 + 2) (EC_50_ = 6.1 nM, E_max_ normalized to TNF = 58.6%). Likewise, ICMs harboring higher valencies in terms of TNFR1 paratopes triggered stronger caspase-3 induction (TNFR1xHER2 ICM11 (4 + 2): EC_50_ = 7.7 pM, E_max_ normalized to TNF = 94.4%, TNFR1xHER2 ICM11 (6 + 2): EC_50_ = 6.1 pM, E_max_ normalized to TNF = 99%).

Furthermore, engineered ICMs elicited TNF-like activation of caspase-1. Interestingly, maximum induction of caspase-1 was very similar between TNFR1xHER2 ICM11 (2 + 2) and the valency-engineered derivatives TNFR1xHER2 ICM11 (4 + 2) as well as TNFR1xHER2 ICM11 (6 + 2) (E_max_ normalized to TNF = 79.6% vs. 82.7% vs. 86.3%). Besides, like for caspase-8 and caspase-3 activation, we noticed again a strong *cis*-targeting effect for TNFR1xHER2 ICM11 (2 + 2), when compared to the non-targeted control TNFR1xHEL ICM11 (2 + 2) (EC_50_ = 46.3 pM vs. 3.8 nM). Essentially, this demonstrates that generated ICMs have a similar caspase-induction profile to the cytokine TNF itself.

We also set out to investigate the consequences of caspase inhibition on cell death induction, harnessing caspase-1 inhibitor (Ac-YVAD-cmk, InvivoGen), caspase-3 inhibitor (Z-DEVD-FMK, R&D Systems), and caspase-8 inhibitor (Z-IETD-FMK, InvivoGen) as well as pan-caspase inhibitor (zVAD-FMK, InvivoGen). To this end, for each of the TNF-like molecules, cell killing in the presence of 50 μM of the respective inhibitor was normalized to cell death induction without the inhibitor compound (relative cell death, Figure 5E; Table 3). Generally, the impact of individual or pan-caspase inhibition on cell death induction was similar for the generated ICMs when compared to (rh) TNF, giving evidence that the underlying mechanisms of eliciting programmed cell death remain largely conserved. Intriguingly, pan-caspase inhibition almost entirely abolished cell death induction for each of the ICMs or TNF, with relative (remaining) cell death induction ranging between 3.9% (TNFR1xHER2 ICM11 (2 + 2)) and 8.1% (TNFR1xHEL ICM11 (6 + 2)). This is highly indicative that the induction of cell death of breast cancer cell line MCF-7 by TNF, as well as the ICMs, is predominantly caspase mediated, i.e., suggestive of apoptosis and pyroptosis. For all individual caspase inhibitors, we observed at least a trend toward the inhibition of cell death. While caspase-1 inhibition only moderately diminished killing (relative cell death ranging from 82.1% to 95.8% when normalized to killing by the respective compound without the addition of the inhibitor), the effect of impaired programmed cell death induction was significantly more pronounced for caspase-3 and caspase-8 inhibition (caspase-3 inhibition: relative cell death ranging from 56.9% to 70.7%; caspase-8 inhibition: relative cell death ranging from 30.9% to 69.8%).

**Table 3:** Remaining cell death induction of MCF-7 cells after treatment with ICMs in the presence of different caspase inhibitors

Samples	Relative cell death [%]			
	pan-caspase inhibition	caspase-1 inhibition	caspase-3 inhibition	caspase-8 inhibition
TNFR1xHER2 ICM11 (2 + 2)	3.9	87.9	65.5	42.8
TNFR1xHER2 ICM11 (4 + 2)	6.9	84.2	65.1	57.4
TNFR1xHER2 ICM11 (6 + 2)	6.9	92.6	70.5	69.8
TNFR1xHEL ICM11 (2 + 2)	4.6	82.1	56.9	30.9
TNFR1xHEL ICM11 (4 + 2)	7.6	91.3	70.7	64.7
TNFR1xHEL ICM11 (6 + 2)	8.1	95.8	70.1	62.1
(rh) TNF	6.1	82.1	62.8	67.2

### Engineered TNFR1xHER2-targeting ICMs decouple NF-κB-dependent pro-survival signaling from cell death induction in HER-2 overexpressing MCF-7 cells

A dual role of TNFR1-signaling has been described with respect to cell survival and killing.^52^ Besides eliciting cell death, TNFR1 agonism might trigger pathways ultimately resulting in gene activation and survival.^53^ One of the key mediators of cell survival is NF**-**κB activation which has been linked to TNF-derived tumor progression of various types of cancer.^54^^,^^55^^,^^56^^,^^57^^,^^58^^,^^59^^,^^60^ We sought to investigate whether generated HER2-targeting TNFR1-agonizing ICMs potentially mediate pro-survival signaling in cancer cells. To this end, we examined NF**-**κB activation in MCF-7 cells in comparison to (rh) TNF. In this regard, (rh) TNF elicited rather strong NF**-**κB activation in a dose-dependent manner (E_max_ = 30.9%, EC_50_ = 9.9 pM, Table S3; Figures 5D; S8D). In contrast to this, HER2-targeted ICMs were highly diminished in their capacity to trigger NF**-**κB activity in MCF-7 cells. While potencies of HER2-targeted ICM derivatives were in the range of or even stronger than (rh) TNF (ranging from EC_50_ = 0.2 pM for TNFR1xHER2 ICM11 (6 + 2) to EC_50_ = 1.0 pM for TNFR1xHER2 ICM11 (2 + 2), maximum NF**-**κB activities were substantially reduced for all sdAb-derived agonists (E_max_ between 5.4% for TNFR1xHER2 ICM11 (2 + 2) and 5.7% for TNFR1xHER2 ICM11 (6 + 2). For example, when normalized to (rh) TNF (Table 2), bivalent TNFR1xHER2 ICM11 (2 + 2) only triggered NF**-**κB activity of 18.6% (TNF-normalized E_max_), while the induction of cell death of MCF-7 cells was fairly TNF-like (82.4%). Interestingly, also hexavalent TNFR1xHER2 ICM11 (6 + 2), which was augmented in terms of killing capacities in comparison to (rh) TNF (E_max_ killing normalized to (rh) TNF = 111.9%), showed substantially attenuated NF**-**κB activation (TNF-normalized E_max_ of NF**-**κB = 20.1%). Consequently, all HER2-targeted valency-engineered ICMs (2 + 2, 4 + 2, 6 + 2) decoupled TNF-like tumor cell death induction from pro-survival signaling in MCF-7 cells. Besides, the non-targeted control ICMs were substantially diminished in terms of potencies for triggering NF**-**κB activity, again corroborating a *cis*-targeting effect, which was evident for all valency-engineered ICM-agonists.

### HER2-specific TNFR1-engaging ICMs elicit substantially reduced untargeted pro-inflammatory cytokine release from PBMCs compared to TNF

**Figure 6:**
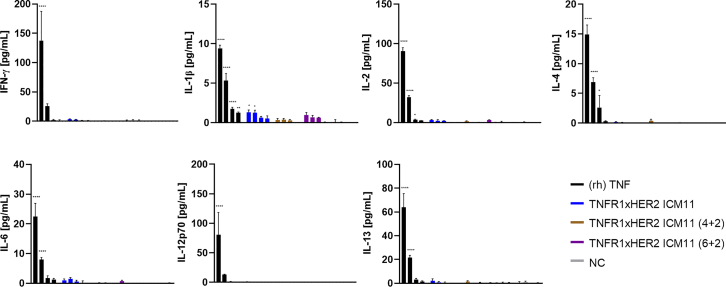
HER2-specific TNFR1-engaging ICMs elicit diminished pro-inflammatory cytokine release in PBMCs compared to (rh) TNF Pro-inflammatory cytokine release of human PBMCs triggered by (rh) TNF in comparison to TNFR1xHER2 ICMs. Human PBMCs were stimulated with 100 nM, 10 nM, 1 nM, and 0.1 nM of (rh) TNF or ICMs for 24 h. Cytokine release was quantified with MSD proinflammatory panel 1 (human) Kit (MSD). Mean values ±SEM of four independent experiments are shown. ∗∗∗∗*p* < 0.0001,∗∗∗*p* < 0.001, ∗∗*p* < 0.01, and ∗*p* < 0.05 calculated by utilizing two-way ANOVA multiple analyses and Bonferroni test.

As a pro-inflammatory cytokine, TNF is one of the main inducers of inflammation.^61^ As such, TNF triggers the production of other pro-inflammatory cytokines, for instance, IL-1 or IL-6, which, in part, might explain significant toxicities associated with systemic TNF therapy.^3^^,^^62^^,^^63^ As a surrogate for systemic delivery, we aimed at assessing the release of several pro-inflammatory cytokines from peripheral blood mononuclear cells (PBMCs) treated either with (rh) TNF or HER2-specific TNFR1-addressing ICMs in an unconditional manner. To this end, human PBMCs were either incubated with (rh) TNF or TNFR1xHER2-specific ICMs using various concentrations (100 nM, 10 nM, 1 nM, and 0.1 nM) for 24 h. Afterward, we specifically looked at the production, i.e., levels of IFN-γ, IL-1β, IL-2, IL-4, IL-6, IL-12, as well as IL-13 in the supernatant. This revealed for all cytokines tested, that engineered TNFR1xHER2-specific ICMs triggered significantly lower levels of production from PBMCs compared with (rh) TNF (Figure 6; Table 4). At concentrations of 100 nM and 10 nM, (rh) TNF for instance, elicited the IFN-γ production of 137.6 pg/mL and 25.8 pg/mL, respectively. In contrast to this, TNFR1xHER2 ICM11 derivatives only marginally induced IFN-γ release in the low single-digit pg/mL range at all analyzed concentrations. Even for IL-1β, that was the only pro-inflammatory cytokine for which we observed significant release induced by TNFR1xHER2 ICM11 (2 + 2) (1.3 pg/mL at 100 nM and 1.3 pg/mL at 10 nM), levels were reduced compared to (rh) TNF (9.2 pg/mL at 100 nM and 5.3 pg/mL at 10 nM). For the other cytokines scrutinized, differences in elicited production by the engineered TNFR1xHER2 ICM11 molecules in comparison to (rh) TNF were appreciably more pronounced. While (rh) TNF caused reasonable release of IL-4 and IL-6 (IL-4 release of 14.9 pg/mL at 100 nM and 6.9 pg/mL at 10 nM, IL-6 production of 22.5 pg/mL at 100 nM and 8.0 pg/mL at 10 nM), we were almost unable to detect release of these pro-inflammatory cytokines for some of the bispecific agonists. Finally, also for induced IL-2, IL-12, and IL-13, cytokine release levels were substantially different. Here, (rh) TNF triggered the release in the higher double-digit pg/mL range at a concentration of 100 nM, whereas generated bsAbs only induced a mild release in the low single-digit pg/mL range. Essentially, in direct comparison to (rh) TNF, this might be envisioned to translate into a better safety profile when administered systemically.

**Table 4:** Pro-inflammatory cytokine release of human PBMCs induced by HER2xTNFR1 ICMs and (rh) TNF

Samples	Pro-inflammatory cytokine release						
	IFN-γ [pg/mL]	IL-1β [pg/mL]	IL-2 [pg/mL]	IL-4 [pg/mL]	IL-6 [pg/mL]	IL-12p70 [pg/mL]	IL-13 [pg/mL]
(rh) TNF							
100 nM	137.6	9.4	90.5	14.9	22.5	80.5	63.8
10 nM	25.8	5.3	32.1	6.9	8.0	13.2	21.5
1 nM	2.1	1.7	3.8	2.6	1.7	1.5	3.1
0.1 nM	1.5	1.3	2.3	0.3	1.2	0.5	1.5
TNFR1xHER2 ICM11 (2 + 2)							
100 nM	3.1	1.3	2.9	0.2	1.0	0.8	2.1
10 nM	2.4	1.3	2.0	0.1	1.5	0.3	0.9
1 nM	0.9	0.6	2.2	ND	0.7	0.1	0.6
0.1 nM	0.9	0.5	ND	ND	0.4	ND	ND
TNFR1xHER2 ICM11 (4 + 2)							
100 nM	0.6	0.4	0.1	ND	ND	ND	ND
10 nM	0.7	0.4	1.6	0.4	0.1	0.1	1.4
1 nM	0.6	0.3	ND	ND	0.3	ND	0.1
0.1 nM	0.7	ND	0.3	ND	0.2	ND	0.5
TNFR1xHER2 ICM11 (6 + 2)							
100 nM	1.2	1.0	3.2	ND	0.7	0.2	0.4
10 nM	1.4	0.7	0.2	0.1	0.1	ND	0.3
1 nM	1.3	0.6	2.8	ND	ND	0.1	0.6
0.1 nM	0.8	0.1	ND	ND	0.1	ND	0.4

### Structural modeling of TNFR1-ICM11 complexes

**Figure 7:**
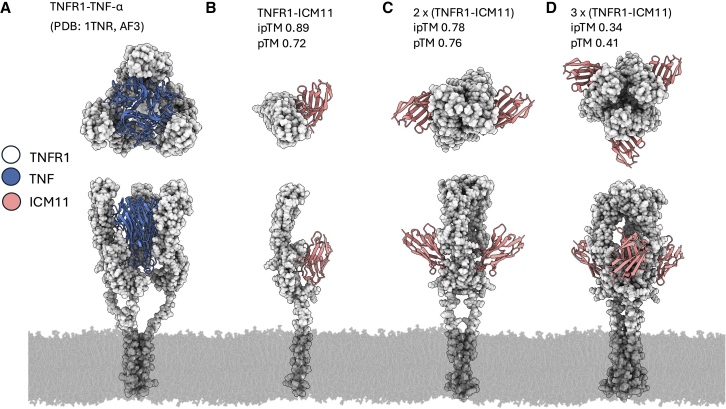
AlphaFold3 models of TNFR1-ICM11 complexes (A) Native TNFR1–TNF reference structure for epitope orientation (adapted from PDB: 1TNR). The following contact residues defines TNF binding site: Lys18, Ser49, His52, Trp93, Glu95, Arg132, Lys143, Lys144, Glu42, Ser43, His55, Cys56, Cys59, Ser60, Lys61, Arg63, Lys64, Glu65, and Met66. (B–D) AF3 models for 1:1, 2×, and 3× TNFR1-ICM11 assemblies. The AF3 confidences are indicated in the figure: ipTM provides confidence in the interface quality, and pTM summarizes the overall complex topology. The resulting contact residues of the 1:1 complex are: Arg63, Glu65, His91, Tyr92, Trp93, Glu95, Asn96, Gln99, Phe101, Lys118, Arg132, Glu133, Glu135, Glu147, and Lys150. Shared TNFR1 hotspot residues shared by both TNF and ICM11: Arg63, Glu65, Trp93, Glu95, Arg132. Interface/contact residues were computed via Molecular Operating Environment (MOE) software.^65^

To rationalize ICM11 agonism and valency-dependent potency/selectivity trends, we modeled TNFR1-ICM11 assemblies using AF3.^64^ We first compared the AF3 models to the native TNFR1-TNF reference shown in Figure 7A to orient the TNF epitope on TNFR1 (PDB 1TNR was used). The top 1:1 TNFR1-ICM11 model (Figure 7B) predicts an interface on TNFR1 that significantly overlaps the native TNF binding site, consistent with our epitope binning that places the agonists in the TNF bin. For completeness, we report AF3 template modeling (TM) confidence metrics for each model in Figure 7: (B) TNFR1-ICM11 ipTM = 0.89, pTM = 0.72; (C) 2×(TNFR1-ICM11) ipTM = 0.78, pTM = 0.76; (D) 3×(TNFR1-ICM11) ipTM = 0.34, pTM = 0.41. The ipTM is AF3’s interface predicted TM score that reflects confidence in the relative positioning of subunits in a complex, whereas pTM is the predicted TM score summarizing overall complex topology. In line with the SEC/SDS evidence for a stable TNFR1-ICM11 complex (Figures 7B and S8), the modeled binding region includes shared hotspot residues on TNFR1 (Arg63, Glu65, Trp93, Glu95, and Arg132) while the full contact lists are provided in the caption of Figure 7. Multi-body assemblies comprising two and three copies of TNFR1-ICM11 retain this footprint qualitatively (Figures 7C and 7D). The 2×(TNFR1-ICM11) assembly (ipTM 0.78, pTM 0.76) illustrates a plausible geometry for multivalent clustering proximal to the membrane, whereas the 3× assembly (ipTM 0.34, pTM 0.41) suggests a potential tri-cluster arrangement but is interpreted cautiously given its lower model confidence. Together, these structures suggest a mechanistic rationale for the increased potency observed upon raising TNFR1 valency (from 2 + 2 to 4 + 2) and the limited additional EC50 benefit at 6 + 2 alongside erosion of the *cis*-selectivity window. The models also visualize how non-productive monovalent occupancy at high ligand excess could compete with multivalent clustering, consistent with the “hooking” effect seen for bispecific agonists.

## Discussion

TNF is a highly pleiotropic cytokine involved in a diverse set of processes such as inflammation, host defense, cell survival, and proliferation, but it can also induce cell death, for instance, via apoptosis and pyroptosis.^1^^,^^6^ TNF can bind to two cognate receptors, TNFR1 as well as TNFR2. While TNFR1 is more ubiquitously expressed, TNFR2 expression is mainly restricted to the immune cell compartment. As such, TNFR2 functions, for instance, as a co-stimulatory receptor on conventional T cells but also as an activating receptor on Tregs.^9^^,^^10^^,^^11^ Especially, in settings of chronic inflammation, TNFR2 expression is significantly upregulated on tumor-infiltrating Tregs, where it triggers activation, proliferation, as well as phenotypic stability.^9^ In the case of TNFR1, it was shown in murine models that TNF-induced tumor apoptosis was a key mechanism of tumor cell killing by effector T cells.^66^^,^^67^ The duality of TNF for cancer treatment also becomes evident when looking at clinical trials, where either TNF blockade combined with immune checkpoint inhibition is currently being explored or the targeted delivery of TNF to the tumor microenvironment in the form of immunocytokines.^1^^,^^28^ Furthermore, TNFR2 antagonists as well as agonists are being investigated in clinical development for cancer treatment.^30^^,^^31^^,^^32^^,^^33^^,^^34^

In the present work, we aimed at exploring whether targeted TNFR1 agonism can be harnessed for the preferential induction of cell death in tumor cells. For this, we focused on a bsAb approach harnessing camelid-derived sdAbs as targeting entities.^68^ Antibody therapeutics, including bi- or multispecific approaches,^39^^,^^69^ have revolutionized disease treatment, as evidenced by the fact that approximately 200 different Abs have been approved by different health authorities (YAbS database: https://db.antibodysociety.org/., accessed on December 22, 2025).^70^ Due to their simple architecture, ease of generation as well as multiple reformatting options, camelid-based sdAbs emerged as auspicious building blocks for the generation of multifunctional biologics.^68^^,^^71^^,^^72^ To this end, camelids were immunized with the ECD of (rh) TNFR1, and by using yeast surface display as a platform technology,^37^ we were able to select for a set of TNFR1-specific sdAbs. Subsequently, those were reformatted into a bispecific format enabling bivalent engagement of both targets (2 + 2) by employing a HER2-directed VHH as a second paratope for *cis*-delivery. In reporter cell assays, five of those molecules triggered receptor agonism in a meaningful way, with a range of activities in terms of potencies and efficacies. In this regard, we have not been able to clearly decipher the underlying reason for their differences in agonism capacities, since affinities of generated TNFR1-specific VHHs were all in the same range, and the binders also targeted similar epitopes on TNFR1. To provide structural context, we compared AF3 models of the TNFR1–ICM11 VHH complex to the native TNF-TNFR1 reference and to our biochemical evidence of a stable TNFR1–ICM11 assembly by analytical SEC with SDS-PAGE co-elution. The 1:1 model places ICM11 on a TNF-like epitope on TNFR1, consistent with the epitope-binning result that all agonists fall into the TNF bin, supporting the notion that differences in agonism among clones with similar affinities and bins may arise from geometry (approach angle/footprint) rather than affinity alone, offering a mechanistic rationale for the functional heterogeneity observed in reporter assays.

HER2-targeting enabled the preferential delivery of the TNFR1 agonism to tumor cells, since HER2-directed TNFR1-based ICM (ICM11) elicited very potent killing of HER2 overexpressing cancer cells, nearly similar to (rh) TNF, whereas the corresponding untargeted control molecule was heavily attenuated by more than 200-fold. While we were able to augment killing features by increasing valencies for the sdAb directed against TNFR1, it must be emphasized that this also resulted in a strongly decreased *cis*-targeting score. Eventually, this might result in a more severe systemic TNFR1 activation. In recent years, *cis*-delivery of cytokine-derived immunotherapies emerged as a promising strategy in order to enhance anti-tumor efficacy and to reduce toxicity.^73^ In this regard, it has been shown that IL-2 can be delivered in *cis* by fusing the cytokine payload to a PD-1, CD8, or T cell receptor-specific paratope.^74^^,^^75^^,^^76^ Additionally, *cis*-targeting has been described for IL-12, IL-15, and IL-21.^77^^,^^78^^,^^79^ While primarily, the cytokine payload is intended for the preferential activation of immune cell subsets, such as T cells, our study demonstrates that the cytokine payload in the form of a cytokine mimetic can be directed toward tumor cells by incorporating a VHH targeting HER2. For the TNFR1 agonism stimulus, HER2 blockade is not mandatory, since the receptor mainly functions as an anchor for payload delivery. Nonetheless, the incorporation of a HER2 blocker can be envisioned to enable a dual mode of action, resulting in even more pronounced efficacy.^80^^,^^81^

Interestingly, killing capacities triggered by the herein generated ICMs were accompanied by the TNF-like induction of caspase-8, caspase-3, as well as caspase-1 activity. Related to this, the addition of a pan-caspase inhibitor almost entirely abolished programmed cell death induction, clearly indicating apoptosis and pyroptosis as the main underlying mechanisms of tumor cell killing. Fascinatingly, using MCF-7 cancer cells, the ability to induce NF-κB pro-survival signaling was substantially compromised for the different valency engineered ICMs when compared with (rh) TNF. Hence, it is tempting to speculate that this might result in decoupled programmed cell death induction from pro-survival signaling, which would be beneficial in the tumor context.^53^^,^^54^^,^^55^^,^^56^^,^^57^^,^^58^^,^^59^^,^^60^ Another advantageous feature of the herein engineered ICMs relied on their very limited induction of pro-inflammatory cytokines from PBMCs when applied in an unconditional manner, i.e., without HER2-expressing target cells. Whereas (rh) TNF triggered the pronounced PBMC-derived secretion of several cytokines such as IFN-γ, IL-1β, IL-2, IL-4, IL-6, or IL-12, this pro-inflammatory attribute was substantially reduced for all valency-engineered TNFR1-based ICMs, which might provide a safety benefit when applied systemically. Notwithstanding, it has been shown that the TNF:TNFR1 axis might have tumor-promoting effects in the tumor microenvironment, ultimately resulting in myeloid cell recruitment.^82^ Furthermore, TNFR1 agonism by TNF induced dendritic cell apoptosis *in vitro,* thereby further enabling tumor growth.^82^ While it can be speculated that *cis*-delivery of TNFR1 agonism to the intended cell population, i.e., tumor cells, would substantially diminish the detrimental effects of systemic TNFR1 agonism, further studies are inevitable to confirm this.

VHH-based cytokine receptor agonists, referred to as cytokine mimetics or surrogate agonists,^83^ have been generated to mimic the function of various cytokines in a tailor-made way, such as IL-2,^84^ type I interferon,^85^ IL-18,^40^ or IL-12.^42^^,^^86^ This study adds TNF to the different cytokines that can be mimicked in a very engineered way, i.e., by agonizing only TNFR1 and by generating immunocytokine versions of the mimetics (named ICMs) via fusion to a paratope directed against a tumor-associated antigen for preferential payload delivery.

Within recent years, antibody-based agonism of TNFR superfamily members has emerged as a promising approach in terms of therapeutic interventions. Mitazalimab, for instance, is a CD40 agonist, which is currently being investigated in clinical trials in patients with solid cancers.^87^^,^^88^ Moreover, targeting death receptor 4 (DR4) and death receptor 5 (DR5) in an agonistic manner is being exploited for cancer therapy, as reviewed by Dubuisson and Micheau.^89^ In addition, specific agonism of 4-1BB (CD137) in a tumor-targeted manner attracted considerable interest.^39^^,^^41^^,^^90^ Essentially, findings from the present study provide evidence that the selective targeting of TNFR1 when combined with a tumor targeting arm enables efficient programmed cell death induction in a *cis*-targeted manner.

### Limitations of the study

One clear limitation of this study is that the *in vitro* setting cannot fully recapitulate the functional consequences of TNF administration *in vivo*. In the present work, we focused on a mechanistic understanding of TNFR1 engagement by ICMs as well as on functional ramifications caused by antibody engineering. Due to the low sequence identity between human TNFR1 (ECD) and murine TNFR1 (ECD, 65%), the generated human TNFR1-specific sdAbs did not cross-react with the murine counterpart (data not shown). Consequently, further elucidations employing mouse-specific TNFR1 sdAbs are needed in order to substantiate whether this TNFR2-decoupled targeted delivery of a tailor-made TNF functionality to tumors might provide a benefit for therapy.

## Resource availability

### Lead contact

Further information and requests for reagents and resources should be directed to and will be fulfilled by the lead contact, Stefan Zielonka (stefan.zielonka@tu-darmstadt.de).

### Materials availability

Requests for new materials generated in this paper are to be directed to and will be fulfilled (pending MTA and associated restrictions) by the lead contact.

### Data and code availability

• Data reported in this paper will be shared by the lead contact upon request.
• This paper does not report original code.
• Any additional information required to reanalyze the data reported in this paper is available from the lead contact upon request.

## Acknowledgments

We thank Ramona Gaa, Florian Gross, Hannah Melina Mayer, Kerstin Hallstein, Deniz Demir, Sonja Dreher, Nils Bahl, Christina Bauer, Vanessa Siegmund, and Sigrid Auth for experimental support.

M.S. and P.S. are supported by the 10.13039/501100001659Deutsche Forschungsgemeinschaft (DFG, SFB1423 “Structural Dynamics of GPCR Activation and Signaling,” project number 421152132, subproject Z03 (to MS and PS) and 10.13039/501100001659DFG under Germany’s Excellence Strategy—EXC 311 2008/1 (UniSysCat)—390540038 (Research Unit E) (to PS).

## Author contributions

L.U., B.L., A.H., J.H., E.G., D.E., M.S., P.S., and A.E. performed all experiments. L.U., S.B., A.M., S.K., P.S., L.P., and S.Z. provided analysis and advice. E.G. and A.E. performed in silico analysis. L.U., L.P., and S.Z. conceptually planned and supervised the study. L.U., E.G., and S.Z. wrote the manuscript with contributions from all authors.

## Declaration of interests

L.U., B.L., J.H., E.G., S.B., A.M., A.E., S.K., D.E., L.P., and S.Z. are affiliated with Merck Healthcare KGaA. Besides, this work was conducted in the absence of any further commercial interest.

## STAR★Methods

### Key resources table

**Table:** 

REAGENT or RESOURCE	SOURCE	IDENTIFIER
**Antibodies**		

anti-his mouse monoclonal detection antibody (SureLight® Allophycocyanin)	Abcam	Cat#ab72579; RRID: AB_1267597
murine anti-his detection antibody (Penta His Alexa Fluor 647 Conjugate)	Qiagen	Cat#35370; RRID: AB_3083468
FITC labeled rabbit polyclonal anti-HA antibody	Abcam	Cat#ab1208; RRID: AB_298835
Trastuzumab, Herceptin	Roche	PZN 16753807
mouse anti-human IgG1 Fc Secondary Antibody (Alexa Fluor™ 488)	Invitrogen	Cat#A10631; RRID: AB_2534050
anti-NF-κB p65 (pS529) Alexa Fluor® 488	BD Biosciences	Cat#558421; RRID: AB_647091

**Biological samples**		

Human peripheral mononuclear cells (PBMCs), isolated from fresh whole blood	Inhouse	N/A

**Chemicals, peptides, and recombinant proteins**		

Peptone	Merck KGaA	Cat#1.07213
D(+)-Glucose	Merck KGaA	Cat#1.08337
Yeast extract	BD Biosciences	Cat#288620
Minimal SD Base	Takara Bio	Cat#630411
Tryptophan dropout mix	Takara Bio	Cat#630413
Na_2_HPO_4_	Merck KGaA	Cat#1.06586
NaH_2_PO_4_ x H_2_O	Merck KGaA	Cat#1.06346
Minimal SD Gal (/Raf) (SG-Media)	Takara Bio	Cat#630421
Penicillin-streptomycin	Gibco	Cat#15140-122
Polyethylenglycol 8000 (PEG 8000)	Euroclone	Cat#EMR037001
Expi293F^TM^ Expression Medium	Thermo Fisher Scientific	Cat#A14351
Dulbeccos Modified Eagle Medium (DMEM)	Gibco	Cat#11965092
heat inactivated fetal bovine serum (h.i. FBS)	Gibco	Cat#A5670801
Normocin	Invivogen	Cat#ant-nr-1
Puromycin	Invivogen	Cat#ant-pr-1
Zeocin	Invivogen	Cat#ant-zn-1
fetal bovine serum (FBS)	Gibco	Cat#A5256701
SepMate-50 tubes	StemCell Technologies	Cat#85450
Dimethylsulfoxid (DMSO)	Sigma Aldrich	Cat#D2438
AIM V™ medium	Gibco	Cat#12055
rh TNFR1 ECD	Sino Biological	Cat#10872-H08H
NaClO_4_	Sigma Aldrich	Cat#71853-M
rh TNFR2 ECD	Sino Biological	Cat#TN2-H5227
rh HER2	Sino Biological	Cat#10004-H08H
rh TNF	Acro Biosystems	Cat#TNA-H5228
Phosphate buffered saline (PBS)	Sigma Aldrich	Cat#D8537
Bovine serum albumin (BSA)	Sigma Aldrich	Cat#A9418
rh IL-18	R&D Systems	Cat#9124-IL
staurosporine	Sigma Aldrich	Cat#S5921
caspase-1 inhibitor (Ac-YVAD-cmk)	InvivoGen	Cat#inh-yvad
caspase-3 inhibitor (Z-DEVD-FMK)	R&D Systems	Cat#FMK004
caspase-8 inhibitor (Z-IETD-FMK)	InvivoGen	Cat#inh-ietd
pan-caspase inhibitor (zVAD-FMK)	InvivoGen	Cat#tlrl-vad
Methanol	Sigma Aldrich	Cat#322415

**Critical commercial assays**		

Lymphoprep	StemCell Technologies	Cat#18061
BSAI-HF_V2 20 000 U/mL	BioLabs	Cat#R3733L
Expi293F^TM^ Expression Kit	Thermo Fisher Scientific	Cat#A14635
MabSelect antibody purification chromatography resin	Cytiva	Cat#17543803
Lyzer™-CL GV 0.22 μm centrifugal devices	Merck Millipore	Cat#UFC40GV0S
TSKgel UP-SW3000 column (2 μm, 4.6 × 300 mm)	Tosoh Bioscience	Cat#TSH-23448
Superdex 200 Increase GL 150 column	Cytiva	Cat#28990945
anti-hIgG Fc capture (AHC) biosensors	Sartorius	Cat#18-5060
anti-Penta-HIS (HIS1K) biosensors	Sartorius	Cat#18-5121
Amine Reactive Second-Generation (AR2G) biosensors	Sartorius	Cat#18-5093
Dip and Read™ Amine Reactive Second-Generation (AR2G) Reagent Kit	Sartorius	Cat#18-5095
SYTOX Red Dead Cell Stain	Invitrogen	Cat#S34859
QUANTI-Blue™ medium	Invivogen	Cat#rep-qbs
SYTOX Green Dead Cell Stain	Invitrogen	Cat#S34860
BD Phosflow Lyse/Fix Buffer 5×	BD Biosciences	Cat#558049
FAM-FLICA(R) Caspase 1 Assay Kit	Biomol	Cat#ICT-98
CaspaTag™ Caspase-3 *In Situ* Assay Kit	Merck Millipore	Cat#APT403
CaspaTag™ Caspase-8 *In Situ* Assay Kit	Merck Millipore	Cat#APT408
MSD Proinflammatory Panel 1 (human) Kit	MSD	Cat#K15049D

**Experimental models: Cell lines**		

Human: HEK-Blue™ TNF cells	Invivogen	Cat#HKB-TNFDMYD
Human MCF-7 cells	ATCC	Cat#HTB-22
Human: Expi293F^TM^ cells	Thermo Fisher Scientific	Cat#A14527

**Experimental models: Organisms/strains**		

*Saccharomyces cerevisiae* strain EBY100	Thermo Fisher Scientific	N/A

**Recombinant DNA**		

cDNA from *Lama glama*, *Vicugna pacos*, *Huarizo*	Preclinics	Immunization, RNA isolation & cDNA synthesis was performed at Preclinics
pDisp plasmid	Geneart	Cat#18AASNIC
pTT5 expression vector	Inhouse	N/A

**Software and algorithms**		

BD FACSDiva™ Software	BD Biosciences	RRID:SCR_001456
FlowJo v10.10	Tree Star	RRID:SCR_008520
ForteBio Data Analysis Software 8.0	ForteBio	RRID:SCR_023267
IntelliCyt ForeCyt Software 10.1	Sartorius	N/A
Gen5 Software 3.15.15	BioTek	RRID:SCR_017317
Incucyte Software v2025A	Sartorius	RRID:SCR_026298
molecular modeling software package MOE (Molecular Operating Environment)	Chemical Computing Group Inc.	RRID:SCR_014882
GraphPad Prism version 10.2.1 for Windows	GraphPad Software, Inc.	RRID:SCR_002798
AlphaFold3 webserver	AlphaFold3 webserver	RRID:SCR_025885
PyMOL (The PyMOL Molecular Graphics System)	Schrödinger Inc.	RRID:SCR_000305
ChimeraX, version 1.7.1.	University of California, San Francisco	RRID:SCR_015872

### Experimental model and study participant details

#### Animals for antigen immunization

For animal immunization with antigen, one female llama (*Lama glama*, age: 8 years), one female alpaca (*Vicugna pacos*, age: 17 years) and one male huarizo (*Lama glama x Vicugna pacos*, age: 8 years) were used. Selected antibody diversities were predominant from llama and alpaca library repertoires. A statement about the influence on species, age or sex dependent library diversity cannot be made.

#### Yeast strains and media

For yeast surface display, the *Saccharomyces cerevisiae* strain EBY100 (MATa URA3-52 trp1 leu2Δ1 his3Δ200 pep4HIS3 prb1Δ1.6R can1 GAL (pIU211:URA3)) (Thermo Fisher Scientific) was utilized. Initially, EBY100 were grown in yeast extract–peptone–dextrose (YPD) medium, which contained 20 g/L peptone, 20 g/L dextrose and 10 g/L yeast extract supplemented with 0.1 mg/mL penicillin–streptomycin (Gibco). Following homologous recombination-based cloning, cells harboring library plasmids (pDisp) were cultivated in minimal synthetic defined (SD)-base medium (Takara Bio) with the corresponding dropout mix (Takara Bio) which included all essential amino acids except tryptophan (−Trp) for selection. This medium was supplemented with 5.4 g/L Na_2_HPO_4_ and 8.6 g/L NaH2PO_4_ x H_2_O.

To induce antibody gene expression, cells were transferred to SG dropout medium (−Trp) containing galactose as carbon source instead of dextrose. This medium consisted of SG-base medium (Takara Bio) supplemented with 10% (w/v) polyethylene glycol 8000 (PEG 8000).

#### Cell lines and culture conditions for protein expression

The expression cell line Expi293F (Thermo Fisher Scientific) was cultured in corresponding Expi293 Expression Medium with 120rpm shaking at 37°C and 5% CO_2_. The cell line was not authenticated.

#### Cell lines and culture conditions for functional studies

The HEK-Blue TNF cells (Invivogen, HKB-TNFDMYD) were cultured in Dulbeccos Modified Eagle Medium (DMEM, Gibco), supplemented with 10% heat inactivated fetal bovine serum (FBS, Gibco) and 100 μg/mL Penicillin-Streptomycin (Sigma Aldrich). Cells were passaged twice a week with 0.5 × 10^6^ vc/mL with freshly added antibiotics: 100 μg/mL Normocin (Invivogen), 1 μg/mL Puromycin (Invivogen) and 100 μg/mL Zeocin (Invivogen). The MCF-7 cells were cultured in DMEM (Gibco), supplemented with 10% FBS (Sigma Aldrich). Cells were passaged twice a week with a 1:3 split ratio. All cells were cultivated at 37°C and 5% CO_2_. All cell lines were authenticated by internal cell bank department with STR-analysis and were tested for mycoplasma contamination.

#### Human whole blood samples

Human whole blood samples were provided as blood donations for research purposes by internal occupational health department. Human blood donors were allocated randomly including three males with the ages of 36, 35 and 50 years as well as one 41 year old female. The age and gender of the human blood donor had no impact on the results. Human PBMCs were freshly isolated from healthy donors according to StemCell Technologies’ SepMate PBMC Isolation protocol using SepMate-50 tubes (StemCell Technologies). PBMCs were stored in DMSO-containing freezing medium in liquid nitrogen. For experiments, cells were quickly thawed at 37 °C and handled in AIM V medium (Gibco).

### Method details

#### Camelid immunization

Three camelids were immunized with (rh) TNFR1 ECD (Sino Biological, 10872-H08H). For administration, the antigen was diluted with sterile deionized water according to manufacturer’s recommendation to a stock concentration of 250 μg/mL. The animals were immunized subcutaneously with 0.15 mg antigen emulsified with Gerbu fama adjuvant. The procedure was repeated three times on day 14, day 28 and day 35 using Gerbu S adjuvant. Eight days after the final administration (day 43), a volume of 100 mL whole blood was collected from each animal for subsequent PBMC isolation, RNA extraction and cDNA synthesis. Of note, all processes involving animals were performed at preclinics GmbH, Germany and in accordance with local regulations and animal welfare protection laws. Immunized animals remained alive after final blood collection.

#### Plasmids for yeast surface display and library generation

VHH display libraries in yeast were constructed using homologous recombination-based gap repair cloning. For this, a detailed protocol for specific PCR amplification of VHH fragments and library construction was already described by our group.^36^ Briefly, digestion of the stuffer sequence in the pDisp plasmid with *BsaI* facilitated the genetic fusion of VHH library candidates in-frame to the *N*-terminus of Aga2p through gap repair cloning, enabling the presentation of the sdAb on the yeast cell surface. Additionally, full-length presentation of VHH variants on the yeast cell surface are monitored by an HA epitope that was linked *C*-terminally to Aga2p on the pDisp backbone.

#### Library sorting

EBY100 cells were initially cultivated overnight in SD medium with dropout mix lacking tryptophan (−Trp) at 30 °C and 120 rpm. VHH surface expression was then induced by transferring the cells into SG medium with dropout mix (−Trp) at 10^7^ cells/mL and cultivation for an additional 48 h at 20 °C and 120 rpm. Libraries from each animal were sorted separately to assess specific antigen binding by incubation with 1 μM His-tagged TNFR1. Antigen binding was detected using either an anti-his mouse monoclonal detection antibody (SureLight Allophycocyanin, Abcam, RRID: AB_1267597, diluted 1:20) or a murine anti-his detection antibody (Penta His Alexa Fluor 647 Conjugate, Qiagen, RRID: AB_3083468, diluted 1:20). To detect full-length surface presentation, a FITC labeled rabbit polyclonal anti-HA antibody (Abcam, RRID: AB_298835, diluted 1:20) was applied which allowed a two-dimensional sorting strategy. The FACS procedure was performed on a BD FACSAria Fusion cell sorter (BD Biosciences). Controls including untreated cells, cells incubated only with secondary detection reagents and cells incubated with an unrelated antigen were used for optimal gating for the desired population. After three sorting rounds, the sequencing results of enriched populations were used for a clonotyping strategy to cluster the VHHs based on their CDR3 regions as described previously.^36^^,^^40^

#### Protein expression, purification and analytics

For the expression of bivalent bispecific ICMs (2 + 2, TNFR1xHER2), selected VHH variants directed against TNFR1 were N-terminally fused to the hinge region of an immune effector-silenced IgG1 Fc (LALA-PG), which was further linked by a C-terminal heavy chain (HC) fusion to a VHH directed against HER2, separated by a 15 amino acid linker (3xGly_4_Ser). Same overall architecture was used to generate anti-TNFR1 VHHs in combination with sdAbs directed against hen egg lysozyme (HEL). For multivalent constructs - (4 + 2) & (6 + 2) - identical paratopes of respective TNFR1 VHHs were fused in tandem or tridem arrangement and separated by 15 amino acid linkers (3xGly_4_Ser). Cloning into pTT5 mammalian expression vector^91^ enabled protein expression through transient transfection of Expi293F cells (5 mL or 25 mL), following the manufacturer’s protocol (Thermo Fisher Scientific). Protein containing supernatants from mini- and small-scale production were harvested by centrifugation after 6 cultivation days and purified via MabSelect antibody purification chromatography resin (Cytiva). Following sterile filtration with Lyzer-CL GV 0.22 μm centrifugal devices (Merck Millipore), protein concentrations were measured using a Nanodrop ND-1000 (Peqlab). Protein purities were determined by analytical SEC on a TSKgel UP-SW3000 column (2 μm, 4.6 × 300 mm, Tosoh Bioscience) using an Agilent HPLC 1260 Infinity system. 7.5 μg protein per sample were injected and run at a flow rate of 0.35 mL/min using 50 mM sodium phosphate, 0.4 M NaClO4 pH 6.3 as mobile phase. Finally, complex formation of human TNFR1 with VHH (ICM11) was tested with an analytical SEC. Analytical SEC was performed using a Superdex 200 Increase GL 150 column (Cytiva). TNFR1 and ICM11 were mixed at a 1:2 M ratio (ICM11:TNFR1) in PBS (pH 7.4) and incubated for 1h at 4 °C prior to injection. As controls, TNFR1 and ICM11 were run individually. Multiple fractions across the main peaks were collected and analyzed by 15% SDS-PAGE. All experiments were carried out in triplicates.

#### Biolayer interferometry

For all Biolayer interferometry (BLI) measurements the Octet RED96 system (ForteBio, Pall Life Science) using 25 °C and 1000 rpm agitation settings was employed. The data was fitted and analyzed with ForteBio data analysis software 8.0 using a 1:1 binding model after Savitzky – Golay filtering if needed. To evaluate specific binding to TNFR1, ICM molecules were immobilized on anti-hIgG Fc capture (AHC) biosensors at 5 μg/mL in PBS for 180 s, followed by 45 s sensor rinsing in kinetics buffer (KB; PBS +0.1% Tween 20 and 1% bovine serum albumin, BSA). Association of either TNFR1 or TNFR2 (Acro Biosystems, TN2-H5227) at 250 nM in KB was measured for 180 s followed by a dissociation in KB for 180 s. To validate binding of bivalent bispecific TNFR1xHER2 constructs to (rh) HER2, AHC biosensors were loaded with 5 μg/mL of each antibody in PBS for 180 s, followed by 45 s sensor rinsing in KB. Association of 250 nM HER2 (Sino Biological, 10004-H08H) in KB was recorded for 180 s prior dissociation in KB for 180 s. Competition of Trastuzumab and the exploited VHH-derived paratope was validated by loading of 5 μg/mL HER2 in PBS on anti-Penta-HIS (HIS1K) biosensors for 180 s and biosensor rinsing in KB for 45 s. Association of 250 nM Trastuzumab (Herceptin, Roche) in KB for 300 s was followed by a second association (180 s) of TNFR1xHER2 ICM11 in KB.

To compare kinetic constants of leading five molecules to TNFR1 (KD), 5 μg/mL of the constructs were immobilized on AHC biosensors for 180 s, followed by 45 s sensor rinsing in KB. Association of serial dilutions (200 nM, 1:2 dilutions) of TNFR1 in KB were measured for 300 s followed by dissociation in KB for 600 s.

To evaluate kinetic constant for TNF (Acro Biosystems, TNA-H5228) to TNFR1 (KD), TNF was covalently immobilized on Amine Reactive Second-Generation (AR2G) biosensors at 10 μg/mL in PBS for 300 s. To this end, AR2G biosensors were activated by EDC/NHS complex formation and quenched with 1 M Ethanolamine after immobilization of TNF. Afterward, biosensors were rinsed for 60 s in KB and association of TNFR1 with concentrations from 100 nM to 1.56 nM (1:2 serial dilution in KB) was recorded for 300 s prior dissociation in KB for 600 s.

Competition of leading five agonists and wildtype TNF in binding to TNFR1 were analyzed in epitope binning experiments. For this, HIS1K biosensors were used to load 5 μg/mL TNFRI in PBS for 180 s, followed by 45 s sensor rinsing in KB. Association of 250 nM ICM or TNF in KB for 300 s was combined with a second association (180 s) of a different ICM or TNF at 250 nM. In each experiment, negative controls using an unrelated antibody and an unrelated antigen as well as a baseline association in KB instead of the respective protein were included (data not shown).

#### Surface staining by flow cytometry

All washes and dilutions of cells and samples were performed using flow buffer (1× PBS, 1% BSA). Cells were seeded at 1 × 10^5^ cells/well in round-bottom 96-well plates (Corning) and handled at 4 °C or on ice for entire experiment duration. Cells were stained with 100 nM Trastuzumab or with flow buffer for 1 h. Then cells were washed twice and incubated for 30 min with 200 nM mouse anti-human IgG1 Fc Secondary Antibody (Alexa Fluor 488, Invitrogen, RRID: AB_2534050). Following two more washes, cells were resuspended in a final volume of 100 μL/well flow buffer with SytoxRed (Invitrogen) for dead cell staining and incubated for 15 min at room temperature protected from light. Cells were analyzed using the iQue3 system (Sartorius). Single cells as well as viable cells were gated and overlay histogram plots for comparison of HER2 binding and unstained control were plotted.

#### Human TNF HEK reporter assay

To detect the activation of the downstream signaling pathway the TNF HEK-Blue assay was performed according to the manufacturer’s instructions. In brief, 5×10^4^ cells were seeded into each well of a 96-well plate (Corning) and stimulated with 10 nM and 1 nM of each bivalent bispecific molecule for 24 h at 37°C and 5% CO_2_. Incubation of the HEK-Blue TNF cells with 0.1 nM and 0.01 nM of TNF and IL-18 (R&D Systems, 9124-IL) were used as positive and negative control. For background subtraction cell culture medium only was measured as well. For EC50 determination, 5×10^4^ cells were seeded into each well of a 96-well plate (Corning) and samples as well as positive control TNF were titrated in a 1:10 serial dilution with concentrations ranging from 100 nM to 1 fM. IL-18 as well as a non-agonistic TNFR1 binder (100 nM) were included as negative controls. To test assay setup with HER2 in saturated conditions, assay was additionally performed with a pre-incubation of 1 μM Trastuzumab for 1 h at 37°C and 5% CO_2_ in parallel. After 24 h of incubation 20 μL of cell culture supernatants were mixed with 180 μL QUANTI-Blue medium in a fresh clear 96-well flat-bottom plate (Thermo Fisher Scientific) and incubated for 3 h at 37 °C in a humidity chamber. Optical density was measured at 640 nm using a multi-mode microplate reader (Synergy *NEO*2, BioTek).

#### Cell death induction assay

2.000 MCF-7 cells were seeded into each well of a 384-well plate (Greiner) and stimulated with ICMs or positive control TNF in a 1:3 serial dilution with ranging concentrations from 100 nM to 62.7 fM for 96 h at 37°C and 5% CO_2_. A non-agonistic binder at 100 nM as negative control as well as culture medium only for background subtraction were included. For HER2 blocking, some wells were pre- and co-incubated with 1 μM Trastuzumab. For maximal killing signal a staurosporin (Sigma Aldrich, 30 μM final concentration) control was included. SYTOX Green Dead Cell Stain (Invitrogen, 0.03 μM final concentration) was added to each well to allow for dead cell staining. Plates were analyzed using a live-cell imager (Incucyte, Sartorius) with phase contrast and green fluorescence settings. For time-resolved cell death comparison of TNF, top molecule and multivalent constructs, same assay settings were applied, except using a fixed sample concentration of 1 nM and analyzing the plates every 8 h between 0 and 96 h of incubation. To test the influence of the individual caspases (caspase-1, caspase-3 and caspase-8) on the killing of MCF-7 cells, TNF killing assay was performed with 5 nM ICM or TNF in presence of 50 μM caspase-1 inhibitor (Ac-YVAD-cmk, InvivoGen, inh-yvad) or 50 μM caspase-3 inhibitor (Z-DEVD-FMK, R&D Systems, FMK004) or 50 μM caspase-8 inhibitor (Z-IETD-FMK, InvivoGen, inh-ietd) or pan-caspase inhibitor (zVAD-FMK, InvivoGen, tlrl-vad), respectively. Wells were analyzed after 72 h of incubation.

#### Caspase-1, caspase-3 & caspase-8 activation assays

For intracellular active caspase-1, caspase-3 and caspase-8 detection the FAM-FLICA(R) Caspase 1 Assay Kit (Biomol, ICT-98), the CaspaTag Caspase-3 *In Situ* Assay Kit (Merck Millipore, APT403) and the CaspaTag Caspase-8 *In Situ* Assay Kit (Merck Millipore, APT408) were used primary according to the manufacturer’s instructions but slightly adapted to lower sample volume and cell amount. In brief, 5×10^4^ cells per well were seeded into a 96-well plate (Corning) and stimulated with ICMs or TNF in a 1:3 serial dilution titration with ranging concentrations from 100 nM to 560 fM for 72 h at 37°C and 5% CO_2_ in a humidity chamber. A non-agonistic binder at 100 nM as negative control as well as cell culture medium only for background subtraction were included. After incubation active caspase-1, caspase-3 or caspase-8 were detected intracellular with a carboxyfluorescein-labeled fluoromethyl ketone peptide inhibitor of caspase-1 (FAM-YVAD-FMK), caspase-3 (FAM-DEVD-FMK) or caspase-8 (FAM-LETD-FMK). Therefore, cells were stained with 1× FLICA reagent (150× stock solution) from respective assay kit for 1 h at 37°C and 5% CO_2_. Finally, samples were analyzed with a flow cytometer (iQue3, Sartorius).

#### NF-κB detection in MCF-7 cells by flow cytometry

For NF-κB detection in MCF-7 cells, 5 × 10^4^ cells were seeded into each well of a 96-well round-bottom plate (Corning). Sample dilutions starting at 100 nM to 560 fM were added to the cells and plates were incubated for 40 min at 37°C and 5% CO_2_ in a humidity chamber. After incubation, cells were centrifuged (1.000 rpm for 5 min), washed with pre-cooled washing buffer (1× PBS, 0.5% BSA) and subsequently fixed with 200 μL/well Fixation Buffer (BD Biosciences) at 37°C and 350 rp for 10 min on a plate shaker. Then, cells were washed again twice with washing buffer and permeabilized in 150 μL pre-cooled methanol per well for 30 min on ice. Cells were washed again twice before staining with anti-NF-κB p65 (pS529) Alexa Fluor 488 (BD Biosciences, RRID: AB_647091) or respective isotype control for 45 min on ice. After two additional washes, cells were finally resuspended in 50 μL washing buffer and analyzed on an iQue3 (Sartorius).

#### MSD Multi-Spot Assay for quantification of cytokine release

To evaluate pro-inflammatory cytokine profiles of ICMs vs. TNF in human PBMCs, an MSD Multi-Spot Assay was conducted to quantify cytokine releases. Therefore, 1 × 10^5^ cells per well were seeded into a 96-well plate (Corning) and stimulated with ICMs or positive control TNF at 100 nM, 10 nM, 1 nM and 0.1 nM for 24 h at 37°C and 5% CO_2_. A non-agonistic binder as negative control as well as cell culture medium only for background subtraction were included. After incubation, the released cytokines were measured with MSD Proinflammatory Panel 1 (human) Kit (MSD, K15049D) according to the manufacturer’s protocol. The data were analyzed and concentrations were determined with GraphPad Prism version 10.2.1 for Windows, GraphPad Software, Boston, Massachusetts USA, www.graphpad.com.

#### Molecular modeling, structural visualization and engineering

Structural models of the VHH domains and constant regions of the different bispecific formats were generated using the antibody modeler tool in the molecular modeling software package MOE (Molecular Operating Environment 2020.09: Chemical Computing Group Inc.; 2020). VHHs domains were either directly fused to the constant regions or added via a 3xGly_4_Ser-linker using moe’s protein builder, followed by a conformational search of the linker and an energy minimization of the full constructs. Modeling of the TNFR1-IMC11 complexes were performed using AlphaFold3 webserver.^64^ Visualization of 3D structures and properties were done with PyMOL (The PyMOL Molecular Graphics System, Version 2.3.0 Schrödinger, LLC.) and ChimeraX, version 1.7.1.

### Quantification and statistical analysis

Graphical and statistical analyses were conducted with GraphPad Prism version 10.2.1 for Windows, GraphPad Software, Boston, Massachusetts USA, www.graphpad.com. Dose-dependent curves were fitted using a nonlinear regression curve with three or four parameters, depending on goodness of Fit, to calculate Top and EC_50_ values. P-values were calculated utilizing appropriate ANOVA analyses followed by Bonferroni test as recommended. *p* ≤ 0.05 was regarded as statistically significant (∗∗∗∗*p* < 0.0001,∗∗∗*p* < 0.001, ∗∗*p* < 0.01, ∗*p* < 0.05). The number of n represents the number of individual performed experiments for Figures 2A, 3B, and 5E or the number of different individual PBMC donors for Figure 6 as indicated in the figure legends and shown as mean values ±SEM.
