# Supplementary material for: Antigen-directed single domain antibody-based TNFR1 agonists elicit preferential killing of HER2-overexpressing cancer cells
Source: iScience. 2026 Mar 11;29(4):115327. doi: 10.1016/j.isci.2026.115327 (PMC13053762; doi:10.1016/j.isci.2026.115327)
Supplement: Document S1. Figures S1–S8 and Tables S1–S3 [file mmc1.pdf]

iScience, Volume *29*

Supplemental information

Antigen-directed single domain antibody-based TNFR1 agonists elicit preferential killing of HER2-overexpressing cancer cells

Laura Unmuth, Britta Lipinski, Alicia Hoerr, Julia Harwardt, Enrico Guarnera, Michal Szczepek, Stefan Becker, Andreas Menrad, Patrick Scheerer, Andreas Evers, Simon Krah, Desislava Elter, Lukas Pekar, and Stefan Zielonka

Supplementary Tables

Table S1: Biophysical, biochemical, binding and functional reporter cell properties of the generated bispecific (2+2) surrogate agonists, Related to Figure 2.

Samples	Purity [%]	Yield [mg/L]	HER2 binding	TNFR1 binding	reporter activity		reporter activity (HER2 blocked)	
					EC_{50} [pM]	E_{max} [%]	EC_{50} [pM]	E_{max} [%]
TNFR1xHER2 ICM1	90.3	39.1	P	P	571.2	10.9	10270	4.3
TNFR1xHER2 ICM2	98.7	78.2	P	P	-	-	-	-
TNFR1xHER2 ICM3	98.5	79.4	P	P	-	-	-	-
TNFR1xHER2 ICM4	97.4	44.3	P	P	-	-	-	-
TNFR1xHER2 ICM5	95.3	41.1	P	P	57.8	54.4	939.7	38.9
TNFR1xHER2 ICM6	99.0	61.3	P	P	-	-	-	-
TNFR1xHER2 ICM7	97.9	63.1	P	P	-	-	-	-
TNFR1xHER2 ICM8	96.2	30.4	P	P	-	-	-	-
TNFR1xHER2 ICM9	96.4	53.7	P	O	-	-	-	-
TNFR1xHER2 ICM10	96.7	43.1	P	P	36.9	58.4	853.9	48.9
TNFR1xHER2 ICM11	94.4	67.6	P	P	3.9	84.7	224.0	88.8
TNFR1xHER2 ICM12	98.5	98.3	P	P	127.4	23.7	1123	17.4
TNFR1xHER2 ICM13	96.5	39.1	P	P	59.3	59.0	1119	55.3
(rh) TNF	-	-	-	P	0.3	100	-	-

Table S2: Binding affinities of selected leading five TNFR1 (2+2) surrogate agonists, Related to Figure 2.

Samples	KD [nM]	k_{on} [1/Ms]	k_{dis} [1/s]
TNFR1xHER2 ICM5	12.3	1.80E+05	2.21E-03
TNFR1xHER2 ICM10	12.1	2.57E+05	3.11E-03
TNFR1xHER2 ICM11	15.7	3.32E+05	5.21E-03
TNFR1xHER2 ICM12	21.1	7.41E+05	1.56E-02
TNFR1xHER2 ICM13	23.7	3.31E+05	7.84E-03
(rh) TNF	3.3	1.70E+05	5.62E-04

Table S3: Caspase-1/3/8 & NF-κB activity of bispecific bivalent (2+2) and multivalent (4+2) & (6+2) TNFR1 mimetics, Related to Figure 5.

Samples	caspase-1 activation			caspase-3 activation			caspase-8 activation			NF-κB activation		
	EC_{50} [pM]	E_{max} [%]	E_{max} [% to TNF]	EC_{50} [pM]	E_{max} [%]	E_{max} [% to TNF]	EC_{50} [pM]	E_{max} [%]	E_{max} [% to TNF]	EC_{50} [pM]	E_{max} [%]	E_{max} [% to TNF]
TNFR1xHER2 ICM11 (2+2)	46.3	23.3	79.6	51.2	26.2	62.1	64.2	18.0	48.9	1.0	5.4	18.6
TNFR1xHER2 ICM11 (4+2)	10.6	25.2	82.7	7.7	39.8	94.4	17.8	28.6	82.5	0.4	5.4	19.6
TNFR1xHER2 ICM11 (6+2)	2.7	25.7	86.3	6.1	41.8	99.0	13.1	30.1	89.5	0.2	5.7	20.1
TNFR1xHEL ICM11 (2+2)	3778	18.4	60.5	6136	24.7	58.6	12230	18.0	49.0	1322	2.3	8.3
TNFR1xHEL ICM11 (4+2)	58.6	16.4	61.9	151.6	35.0	82.9	153.2	23.0	62.5	102	3.9	13.6
TNFR1xHEL ICM11 (6+2)	28.4	19.2	63.5	41.6	37.9	89.8	77.2	27.1	73.6	51.2	5.6	20.2
(rh) TNF	49.1	30.2	100	78.4	42.2	100	191.0	36.8	100	9.9	30.9	100

Supplementary Figures

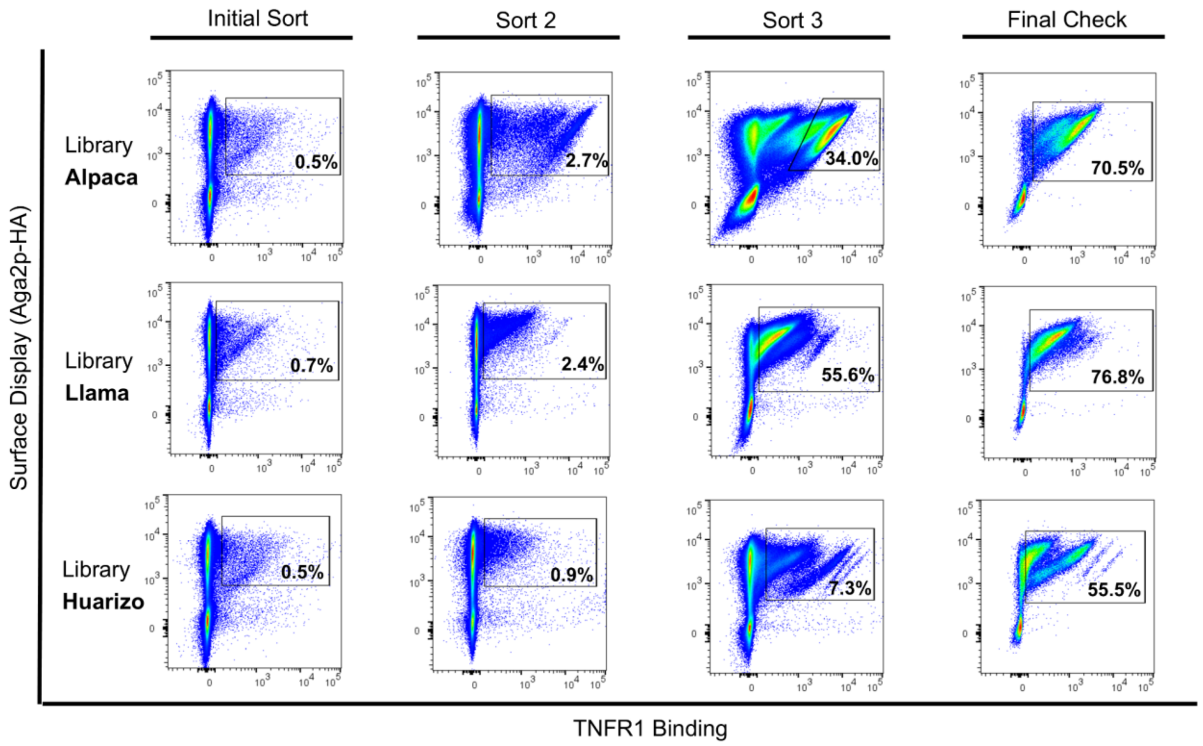

Figure S1: Isolation of camelid-derived TNFR1-specific sdAbs using yeast surface display (YSD), Related to Figure 1. Enrichment of TNFR1 binding VHHs from three immunized camelids by YSD. One library was generated for each specimen (alpaca, llama and huarizo) and sorted separately against 1 µM of (rh) TNFR1 ECD in three rounds to enrich TNFR1-targeting sdAbs. A two-dimensional sorting strategy was applied to select for full-length VHH display in addition to antigen binding. Percentage of cells in sorting gates are shown for each round and each animal. Plots show 5x10^4 events.

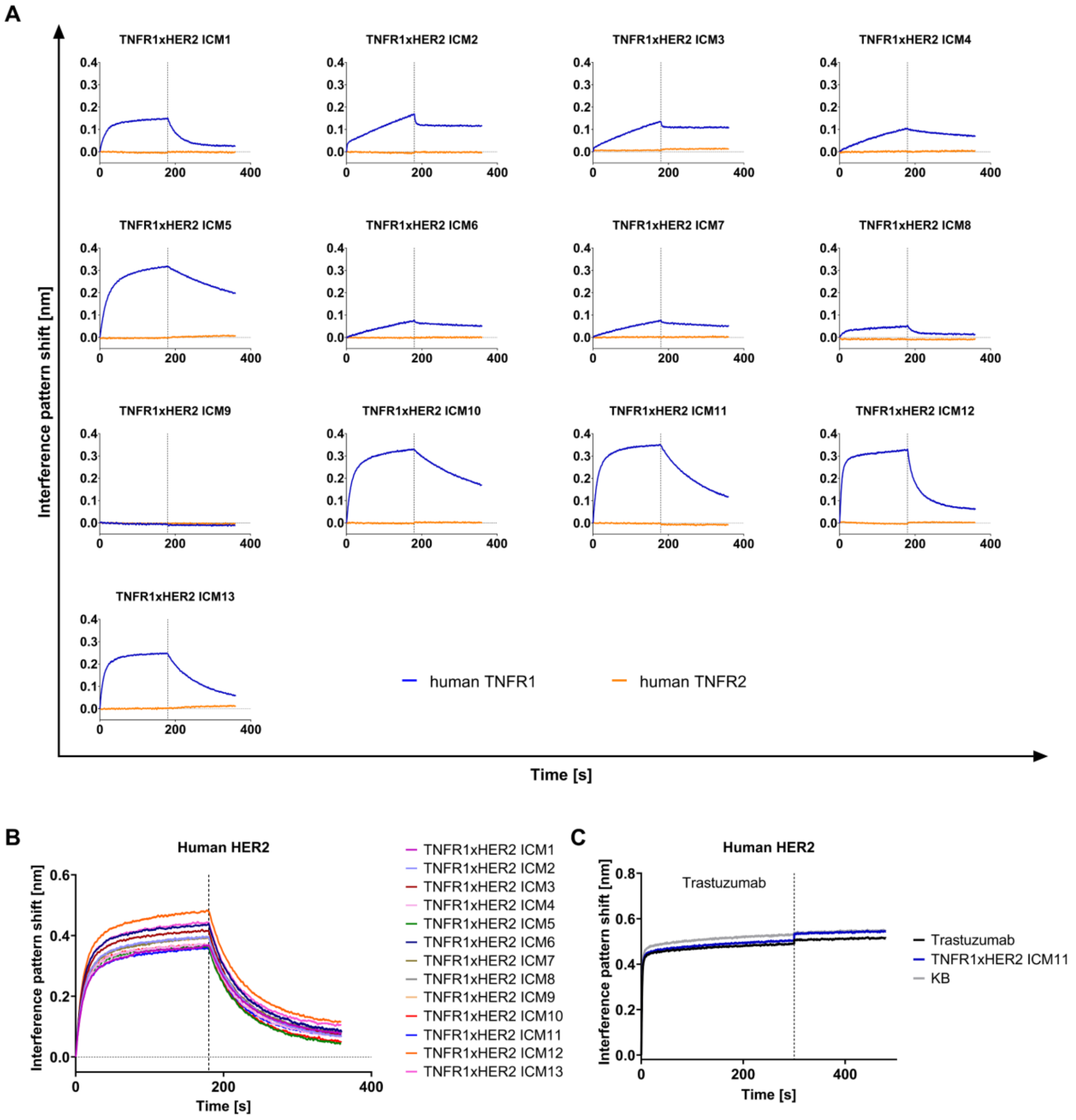

Figure S2: Bispecific (2+2) TNFR1xHER2 ICMs show specific binding to (rh) TNFR1 as well as (rh) HER2 and target a similar epitope on HER2 as Trastuzumab, Related to Figure 2. (A) Binding of TNFR1xHER2 bsAbs to (rh) TNFR1 (blue) or (rh) TNFR2 (orange) as determined by BLI. ICMs were loaded on AHC biosensors at 5 µg/mL for 180 s, followed by association of (rh) TNFR1 or (rh) TNFR2 at 250 nM for 180 s. Subsequently, dissociation was measured for 180 s in kinetics buffer. (B) Binding assessment of bivalent bispecific TNFR1xHER2 constructs to (rh) HER2 by BLI. AHC biosensors were loaded with 5 µg/mL of each antibody for 180 s, followed by association of 250 nM (rh) HER2 for 180 s prior dissociation in kinetic buffer for 180 s. (C) BLI competition assay of Trastuzumab and the exploited VHH-derived HER2 paratope. (rh) HER2 was loaded at 5 µg/mL on HIS1K biosensors for 180 s. First association of 250 nM Trastuzumab for 300 s was followed by a second association of TNFR1xHER2 ICM11 for 180 s.

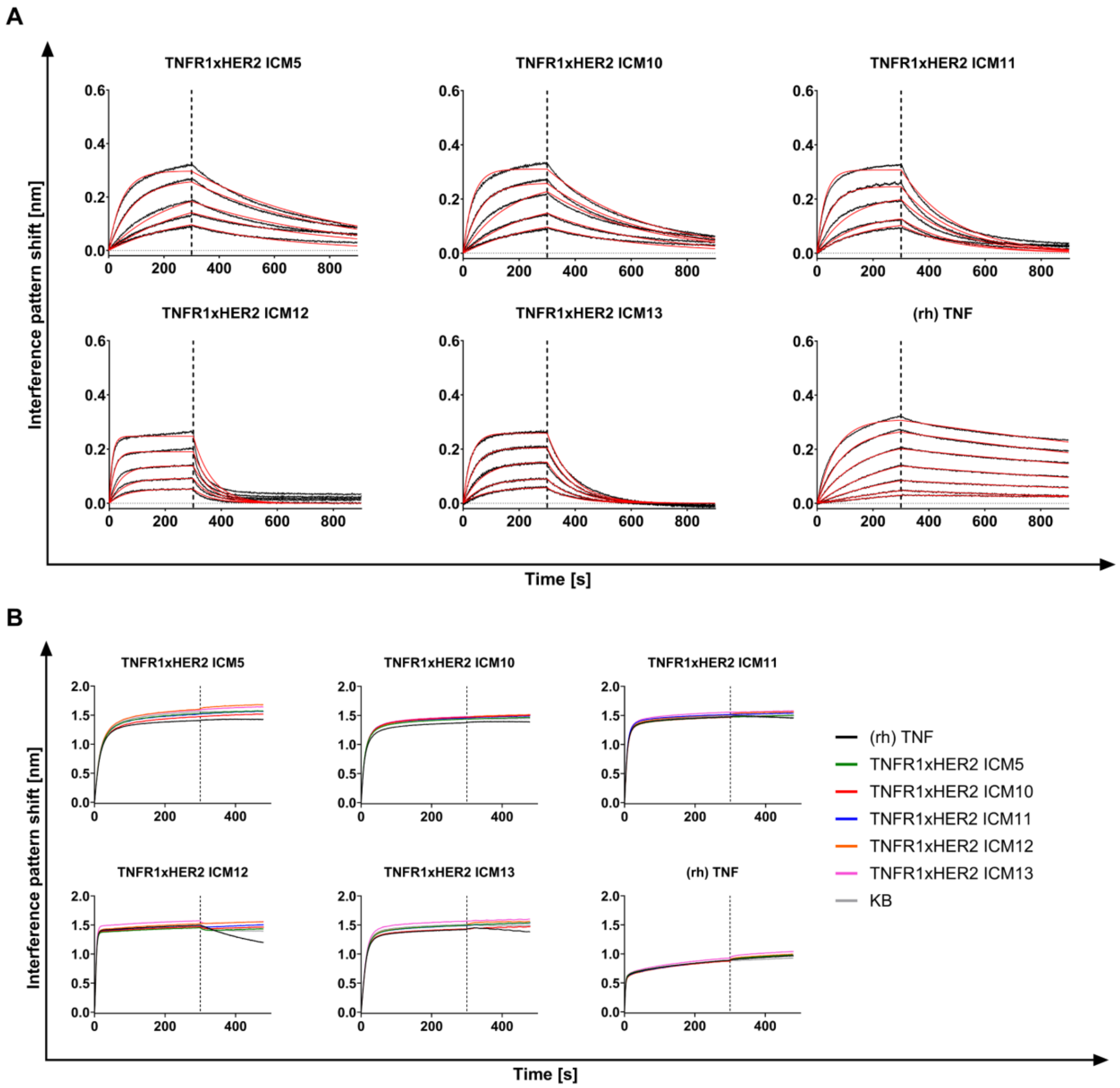

Figure S3: Binding kinetics and epitope binning of bispecific (2+2) TNFR1xHER2 ICMs and (rh) TNF on (rh) TNFR1, Related to Figure 2. (A) Kinetics experiment with BLI to determine affinities to (rh) TNFR1. Constructs were loaded with 5 µg/mL on AHC biosensors for 180 s, followed by association of consecutive dilutions (200 nM, 1:2 dilutions) of (rh) TNFR1 for 300 s and dissociation for 600 s. Affinity of (rh) TNF:TNFR1 interaction was determined by covalent immobilization of (rh) TNF on AR2G biosensors at 10 µg/mL for 300 s via EDC/NHS complex formation and association of (rh) TNFR1 in ranging concentrations from 100 nM to 1.5625 nM for 300 s prior dissociation in kinetic buffer for 600 s. (B) Competition of TNFR1xHER2 agonists and (rh) TNF for binding to (rh) TNFR1 by BLI. HIS1K biosensors were loaded with 5 µg/mL (rh) TNFRI for 180 s, followed by association of 250 nM of respective ICM or (rh) TNF for 300 s combined with a second association of a second antibody or (rh) TNF at 250 nM for 180 s.

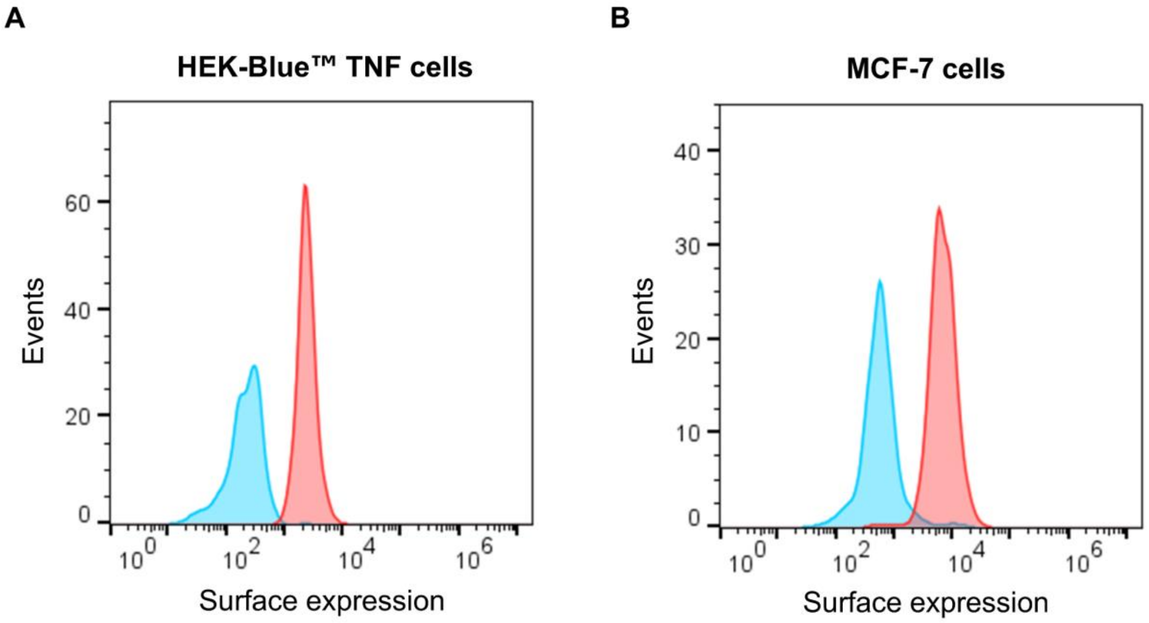

Figure S4: HER2 surface expression of HEK-Blue™ TNF reporter cells and MCF-7 tumor cell line, Related to Figure 2 and 3. (A) Flow cytometry analysis of HER2 on the surface of HEK-Blue™ TNF reporter cells. (B) Flow cytometry analysis of HER2 on the surface of MCF-7 tumor cells. Red areas indicate HER2 expression and blue areas unstained control on respective cells. $1x10^5$ cells were stained with 100 nM Trastuzumab (Herceptin, Roche) or with flow buffer only for 1 hour before an additional 30 min staining with 200 nM detection antibody mouse anti-human IgG1 Fc Secondary Antibody (Alexa Fluor™ 488, Invitrogen, RRID: AB_2534050).

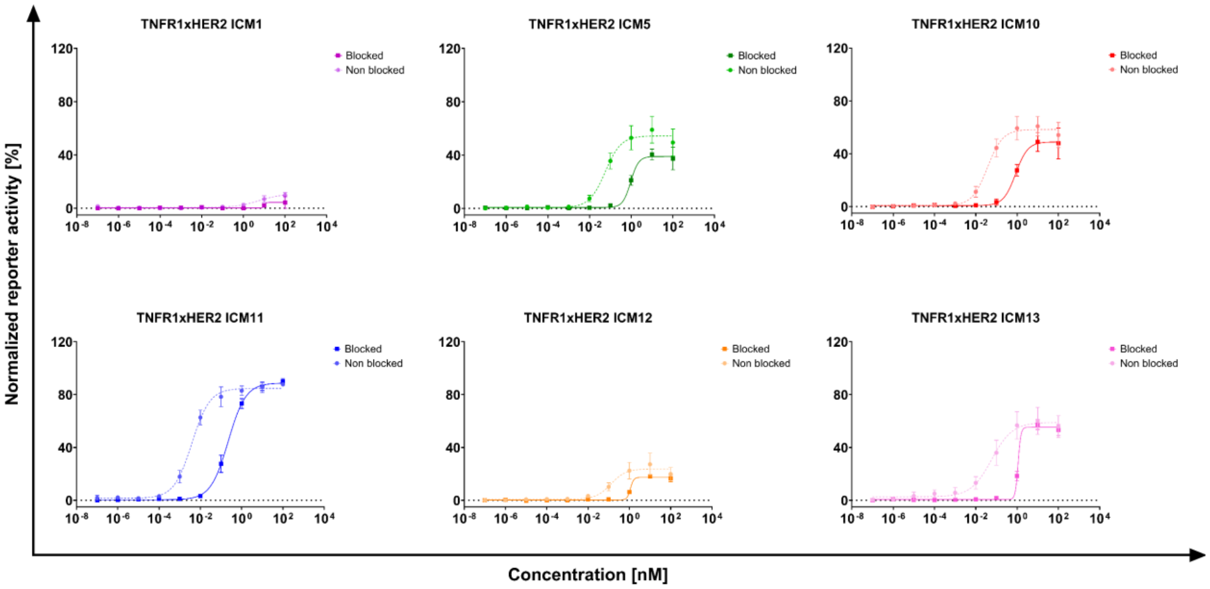

Figure S5: Blocking HER2 on HEK-Blue™ TNF reporter cells reveals *cis*-dependency of TNFR1xHER2 surrogate agonists, related to Figure 2. Reporter cells were pre-incubated with 1 µM Trastuzumab before ICM addition in a dose-dependent manner. After 24 h of incubation secreted embryonic alkaline phosphatase activity was measured via OD_{640}. Reporter activity was normalized to (rh) TNF signal. HER2 blocking by Trastuzumab resulted in significantly attenuated potencies for all ICMs tested. Mean values ± SEM of three independent experiments are shown.

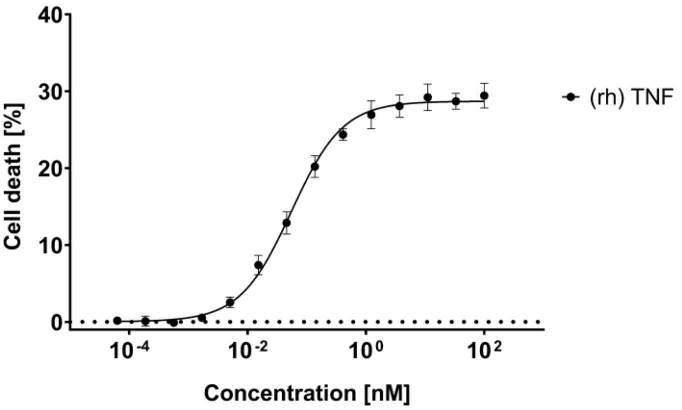

Figure S6: TNF sensitivity of human breast cancer cell line MCF-7 results in cell death induction, Related to Figure 3. Human HER2 expressing MCF-7 cells were stimulated with increasing concentrations of (rh) TNF for 96 h. Killing was monitored by green fluorescence signal with SYTOX Green Dead Cell Stain. After (rh) TNF treatment MCF-7 cells undergo cell death in a dose-dependent-manner. Mean values ± SEM of four independent experiments are shown.

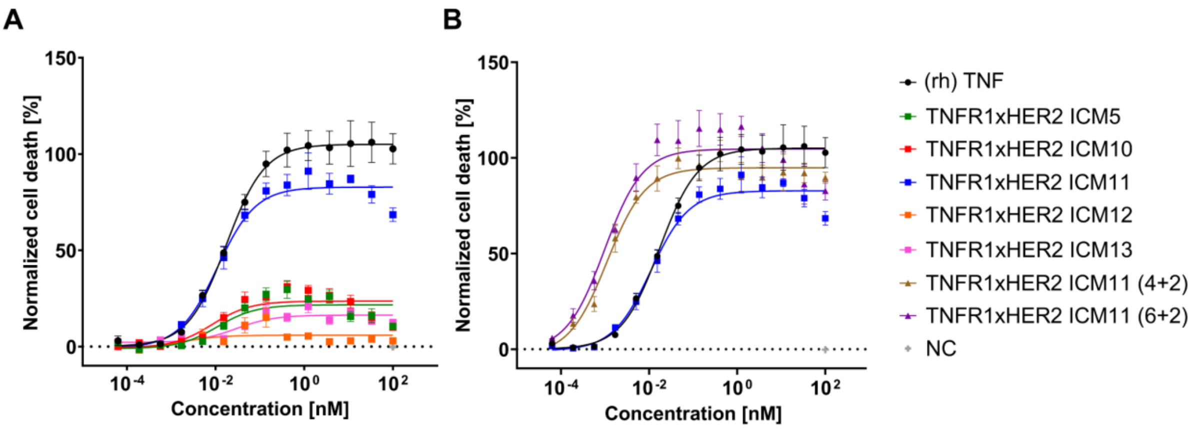

Figure S7: Stimulation of HER2 expressing MCF-7 cells with the full dose-range of compound concentrations is indicating a hooking effect for TNFR1 surrogate agonists induced cell killing, Related to Figure 3 and 4. (A) Killing experiment with tumor cell line MCF-7 and increasing concentrations of bivalent (2+2) TNFR1xHER2 ICMs and (rh) TNF for 96 h. Killing was monitored by green fluorescence signal with SYTOX Green Dead Cell Stain. Cell death induction was normalized to (rh) TNF signal. (B) Killing experiment with tumor cell line MCF-7 and increasing concentrations of multivalent constructs (4+2) TNFR1xHER2 ICM11 and (6+2) TNFR1xHER2 ICM11 in comparison to bivalent (2+2) TNFR1xHER2 ICM11 and (rh) TNF for 96 h. Mean values ± SEM of four independent experiments are shown.

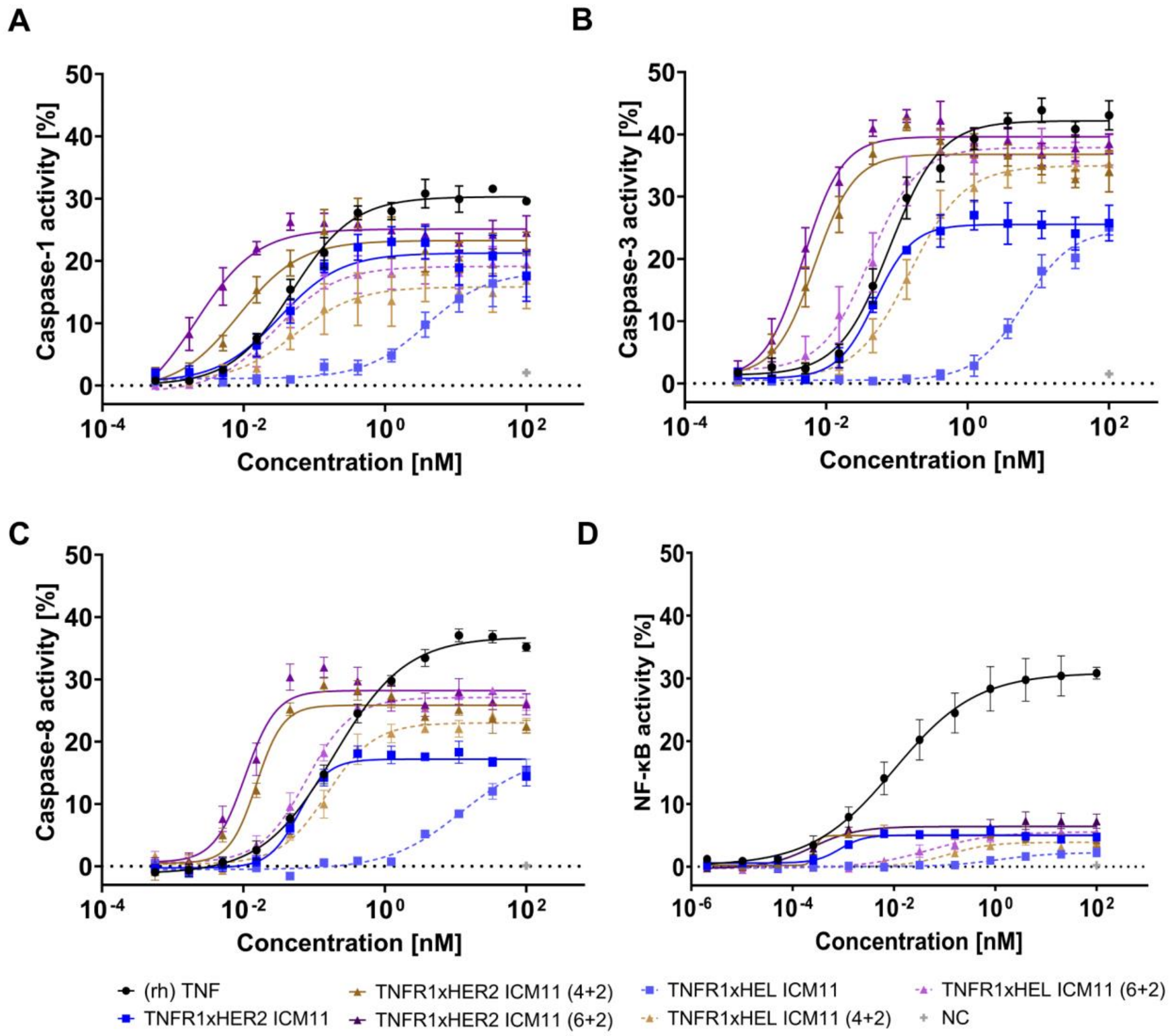

Figure S8: Absolute caspase-1/3/8 and NF-κB activation in HER2 expressing MCF-7 cells by TNFR1 surrogate agonists and (rh) TNF, Related to Figure 5. (A-C) Caspase-1/38 activation assay with tumor cell line MCF-7 and increasing concentrations of TNFR1xHER2 ICMs as well as (rh) TNF for 72 h. Active caspase-1/3/8 was detected intracellular with FAM-FLICA(R) Caspase 1 Assay Kit (Biomol), CaspaTag™ Caspase-3 In Situ Assay Kit (Merck Millipore) and CaspaTag™ Caspase-8 In Situ Assay Kit (Merck Millipore). (D) NF-κB activation assay with MCF-7 cells triggered by increasing concentrations of TNFR1 ICMs for 40 min. NF-κB was stained intracellular with AF488-labeled anti-NF-κB staining antibody after lysis, fixation and permeabilization of cells. Mean values ± SEM of four independent experiments for each figure are shown.
